# Comprehensive bioinformatics analysis and experimental validation: An anoikis-related gene prognostic model for targeted drug development in head and neck squamous cell carcinoma

**DOI:** 10.32604/or.2023.029443

**Published:** 2023-07-21

**Authors:** LIN QIU, ANQI TAO, XIAOQIAN SUN, FEI LIU, XIANPENG GE, CUIYING LI

**Affiliations:** 1 Central Laboratory, Peking University School and Hospital of Stomatology & National Center for Stomatology & National Clinical Research Center for Oral Diseases & National Engineering Research Center of Oral Biomaterials and Digital Medical Devices, Beijing, 100081, China; 2 Department of Dentistry, Xuanwu Hospital Capital Medical University, Beijing, 100053, China; 3 National Clinical Research Center for Geriatric Disorders, Beijing, 100101, China; 4 School of Medicine, Nankai University, Tianjin, 300071, China; 5 Department of Oral and Maxillofacial Surgery, Tianjin Stomatological Hospital, School of Medicine, Nankai University, Tianjin, 300041, China

**Keywords:** Head and neck squamous cell carcinoma, Anoikis, Prognosis, Proliferation, Apoptosis

## Abstract

We analyzed RNA-sequencing (RNA-seq) and clinical data from head and neck squamous cell carcinoma (HNSCC) patients in The Cancer Genome Atlas (TCGA) Genomic Data Commons (GDC) portal to investigate the prognostic value of anoikis-related genes (ARGs) in HNSCC and develop new targeted drugs. Differentially expressed ARGs were screened using bioinformatics methods; subsequently, a prognostic model including three ARGs (CDKN2A, BIRC5, and PLAU) was constructed. Our results showed that the model-based risk score was a good prognostic indicator, and the potential of the three ARGs in HNSCC prognosis was validated by the TISCH database, the model’s accuracy was validated in two independent cohorts of the Gene Expression Omnibus database. Immune correlation analysis and half-maximal inhibitory concentration were also performed to reveal the different landscapes of TIME between risk groups and to predict immuno- and chemo-therapeutic responses. Potential small-molecule drugs for HNSCC were subsequently predicted using the L1000FWD database. Finally, *in vitro* experiments were used to verify the database findings. The relative ARG mRNA expression levels in HNSCC and surrounding normal tissues remained consistent with the model results. BIRC5 knockdown inhibited anoikis resistance in WSU-HN6 and CAL-27 cells. Molecular docking, real-time PCR, cell counting kit-8 (CCK-8), plate clone, and flow cytometry analyses showed that small-molecule drugs predicted by the database may target the ARGs in the prognostic model, inhibit HNSCC cells survival rate, and promote anoikis *in vitro*. Therefore, we constructed a new ARG model for HNSCC patients that can predict prognosis and immune activity and identify a potential small-molecule drug for HNSCC, paving the way for clinically targeting anoikis in HNSCC.

## Introduction

Head and neck cancer (HNC) is the sixth most common malignancy and the leading cause of death and decreased life expectancy worldwide. Oral cavity, oropharynx, and nasopharynx tumors are included in HNC. Over 500,000 fatal HNC cases occurred in 2020, and over one million new cases were diagnosed [1]. Epidemiological studies show that the incidence of HNC is rising annually, with a progressive trend toward a higher incidence in younger people. Squamous cell carcinoma accounts for over 90% of HNC [2]. Although tremendous strides have been made in head and neck squamous cell carcinoma (HNSCC) treatment, the 5-year survival rate remains below 50% since most HNSCC patients have advanced disease at diagnosis [3]. Ninety percent of cancer patients die from metastases [4]; lymph node metastases are the most common in HNSCC and are a key prognostic indicator. The 5-year survival rate is considerably poorer for HNSCC patients who experience lymph node metastases [5,6]. It is essential to develop a predictive model for HNSCC metastasis that occurs before lymph node metastasis to improve patient prognosis.

In general, most cells survive by adhering tightly to the extracellular matrix (ECM) or other cells, and cell death occurs once cells are separated from the ECM. This form of cell death was first named “anoikis” in 1994 [7]. Anoikis is a special type of apoptotic cell death with different cell morphology and biochemical markers from those of ferroptosis, necroptosis, and autophagy [8,9]. Anoikis exhibits some features of apoptosis, mainly depends on the cell-matrix interactions, and plays a critical role in cancer metastasis [7]. Tumor metastasis, by contrast, assumes that cancer cells have evolved resistance to anoikis. When cancer cells are liberated from their cell–ECM and cell–cell adhesion states, they survive, disseminate, and metastasize in the circulatory system by resisting anoikis-induced tumor cell death via autocrine or paracrine pathways [10,11].

The significance of anoikis in HNC is becoming clearer as research and genetic testing procedures develop, and anoikis resistance acquisition has been hypothesized to be a critical step in oral cancer metastasis [12]. The signaling pathways associated with anoikis resistance in HNSCC have been the subject of numerous recent investigations [13,14]. For instance, it has been proposed that the epithelial–mesenchymal transition (EMT) plays a significant role in the development of anoikis resistance in cancer cells [13]. Several scientists have investigated the changes in gene expression that occur after HNSCC cells develop anoikis resistance [14]. However, which genes are essential for HNSCC anoikis resistance and how anoikis-related genes (ARGs) affect patient prognosis remains uncertain. In addition, targeting ARGs may help develop effective drugs that can reduce metastasis in HNSCC patients.

We searched for novel prognostic indicators to explore the role of ARGs in prognosis, predicted potential effective drugs for HNSCC, and introduced new methods for the existing relatively mature comprehensive sequence therapy model. We used bioinformatics methods to construct a high-accuracy ARG-based prognostic model. Subsequently, a small-molecule drug was obtained from the L1000FWD database, and its targeting of the prognostic model genes was validated.

## Materials and Methods

### Data and clinical sample collection

**Figure 1:**
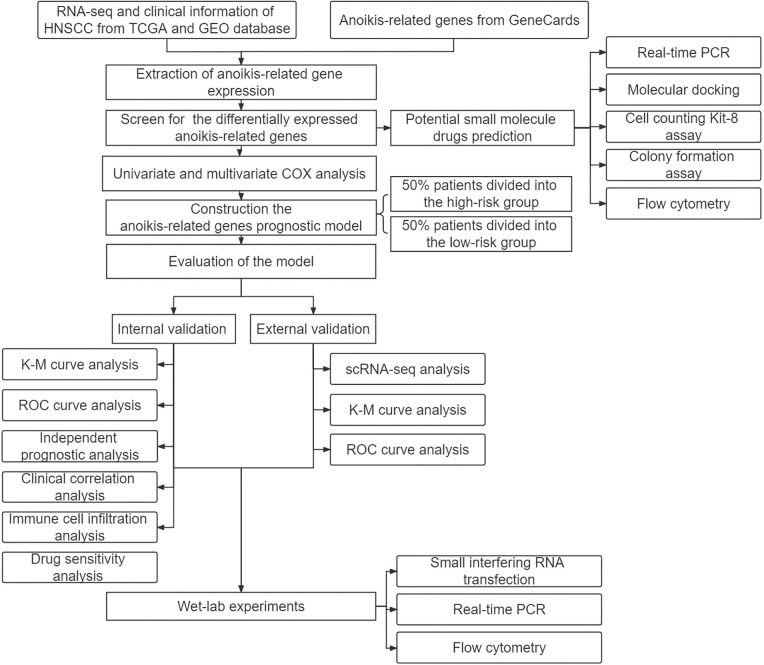
Study design flowchart.

We obtained RNA sequencing (RNA-seq) and clinical information about HNSCC from The Cancer Genome Atlas (TCGA) Genomic Data Commons (GDC) portal (https://portal.gdc.cancer.gov/) and the Gene Expression Omnibus (GEO) database (datasets: GSE27020 n = 99 and GSE41613 n = 76), then removed data with missing survival status, unknown survival time, or survival < 30 days. The single cell RNA sequencing (scRNA-seq) of HNSCC patients was obtained from the TISCH (http://tisch.comp-genomics.org/). (dataset: HNSCC_GSE103322, cell number = 5902). GeneCards was applied to obtain (https://www.genecards.org/) a total of 419 genes (relevance score with anoikis > 0.4) (Suppl. Table S1). Three pairs of HNSCC samples were obtained for real-time PCR (RT–PCR) and were preserved in liquid nitrogen and frozen at −80°C. Our study protocol was approved by the Biomedical Ethics Committee of Peking University Stomatological Hospital. The code used in this study can be found on GitHub (https://github.com/qiulin961028/anoikis-analysis.git), and the study flow chart is shown in Fig. 1.

### Bioinformatics analysis

RNA-seq data of HNSCC patients were analyzed by log2 transformation. Differentially expressed ARGs were subsequently screened and clustered using the R software (4.1.2) LIMMA package. Univariate and multivariate COX analyses were performed to identify HNSCC prognosis-related ARGs for subsequent prognostic model construction.

### Construction and validation of the ARG prognostic model

After the multivariate COX regression analysis, the risk regression coefficients and expressions of each ARG were combined to establish the risk score formula, which led to the prognostic model, calculated as follows:

$$Riskscore=\sum_{i=1}^{n}Coefi\times Xi$$

Coef represents the risk regression coefficient of ARGs, and X represents the ARG expression level. The risk score of each HNSCC patient was measured, and all patients were divided into high- or low-risk groups in accordance with the median risk score. Immediately afterward, the overall survival (OS) outcomes of HNSCC patients were compared between the two risk groups by survival analysis. Receiver operating characteristic (ROC) curves were used to test the accuracy of model predictions. The role of the risk score in predicting prognosis was investigated by univariate and multivariate COX regression analyses.

### Evaluation of immune cell infiltration and prediction of drug therapy response

We quantified differences in immune cell infiltration and immune function in the high- and low-risk groups using ssGSEA. We used the Genomics of Cancer Drug Sensitivity in Cancer (https://www.cancerrxgene.org) to forecast the sensitivity to drug therapies as per the half-maximal inhibitory concentration (IC_50_) in the two groups.

### Potential small-molecule drug prediction

Differentially expressed ARGs were classified into up- or down-regulated groups and imported into the L1000FWD website (https://maayanlab.cloud/L1000FWD/) to obtain outcomes. The top three drug structures are shown on the PubChem website (PubChem.ncbi.nlm.nih.gov).

### Cell culture

The human HNSCC cell lines WSU-HN6 and CAL-27 and the human normal oral keratinocyte epithelial cell line HOK were used in this study. WSU-HN6 and HOK cell lines were obtained from Ninth People’s Hospital, Shanghai Jiao Tong University School of Medicine (Shanghai, China), and the CAL-27 cell line was purchased from American Type Culture Collection (ATCC, Manassas, Virginia, USA). All cells were passaged and preserved at the Central Laboratory of Peking University Hospital of Stomatology and regularly tested to ensure mycoplasma negativity. The methods for detecting mycoplasma in cells were conducted as described previously [15]. All cells were cultured in high-glucose Dulbecco’s Modified Eagle Medium (DMEM) (WSU-HN6 and CAL-27) or Roswell Park Memorial Institute (RPMI) 1640 medium (HOK) (Gibco, Carlsbad, CA, USA) containing 10% fetal bovine serum (Gibco, Carlsbad, CA, USA) and 1% penicillin/streptomycin solution (Solarbio Science & Technology Co., Ltd., Beijing, China) at 37°C and 5% CO_2_.

### Small interfering RNA transfection

The cells were cultured in a 6-well plate at a density of 2.0 × 10^5^ cells/well, and two groups were established: the control group (si-NC) and the interference group (si-BIRC5). According to the recommended protocol, the working concentration of siRNA was 50 nM, and the Lipo8000 (Beyotime Biotechnology Co., Ltd., Shanghai, China) transfection reagent was used. After 24–72 h of culture, the cells were collected for follow-up experiments. The expression of ARG after knockdown was determined by RT–PCR. The siRNAs were synthesized by Tsingke Biotechnology Co., Ltd., China, with the following sequences: si-BIRC5: CCGCATCTCTACATTCAAGAA.

### Real-time PCR

Total RNA was extracted from cells and tissues using TRIzol (Beyotime Biotechnology Co., Ltd., Shanghai, China) and reverse transcribed into cDNA using a Prime Script^™^ RT Kit (TaKaRa Biotechnology Co., Ltd., Tokyo, Japan). The cDNA template was subsequently amplified by RT–PCR using SYBR Green qPCR Master Mix (Abclonal Technology, Wuhan, China). GAPDH was used as an internal reference, and relative mRNA expression was measured by the 2^–ΔΔCT^ method. All primers were purchased from Sangon Biotech (Shanghai, China). The primer sequences are shown below:

GAPDH:

Forward 5′-GCACCGTCAAGGCTGAGAAC-3′,

Reverse 5′-TGGTGAAGACGCCAGTGGA-3′;

CDKN2A:

Forward 5′-CCGAATAGTTACGGTCGGAGG-3′,

Reverse 5′-CACCAGCGTGTCCAGGAAG-3′;

BIRC5:

Forward 5′-TCTGTCACGTTCTCCACACG-3′,

Reverse 5′-GACCTCCAGAGGTTTCCAGC-3′;

PLAU:

Forward 5′-GAGTGCGCTCTTGCTTTGAC-3′,

Reverse 5′-GTGGATGGAATCCGGAGGAC-3′.

### Molecular docking

Molecular docking was performed to analyze the binding capacity of radicicol and ARGs. The PubChem database (https://pubchem.ncbi.nlm.nih.gov/) was accessed to download the radicicol structure, and the protein structure was obtained from the Protein Data Bank (PDB; http://www.rcsb.org/). Hydrogen atoms were added, charges were calculated, and charges and bonds on small molecules were adjusted using AutoDockTools (version 1.5.6), and the data were stored in a PDBQT file. Vina was used to calculate the binding energy, and PyMOL (version 4.6.0) was used to visualize the optimal binding model.

### Cell counting kit-8 assay

Two HNSCC cell lines were inoculated in 96-well plates at 100 μL per well (3000 cells/well) and incubated (37°C and 5% CO_2_). Cell viability was determined using the cell counting kit-8 (CCK-8) assay (Solarbio Science & Technology Co., Ltd., Beijing, China). Ten microliters of CCK-8 solution was added to each well at the appropriate time point (24, 48, and 72 h), and the plate was incubated for 2 h in the dark. The optical density (OD) of each well was measured at 450 nm using a microplate reader (Tecan, Männedorf, Zürich, Switzerland), and cell viability was calculated as follows: cell viability = [(experimental wells’ OD − blank wells’ OD)/(control wells’ OD − blank wells’ OD)] × 100%)].

### Colony formation assay

Two HNSCC cell lines were inoculated in 6-well plates at 1000 cells/well, and 2 mL of medium was added to each well. The culture medium was changed every two days, and the culture was terminated when cell clones were visible to the eye. The cells were washed with phosphate-buffered saline (PBS), fixed with 4% paraformaldehyde (Beyotime Biotechnology Co., Ltd., Shanghai, China) at 4°C for 15 min, and then stained with 0.1% crystal violet (Beyotime Biotechnology Co., Ltd., Shanghai, China) for 10 min at room temperature.

### Flow cytometry

The treated HNSCC cells were collected in 1.5 mL centrifuge tubes, resuspended in precooled 70% ethanol, and fixed overnight at 4°C. After rinsing twice with precooled PBS, 25 μL of PI, 500 μL of staining buffer, and 10 μL of RNase A (Beyotime Biotechnology Co., Ltd., Shanghai, China) were added to each sample for 30 min at 37°C, or samples were stained with 5 μL of Annexin V-FITC and 5 μL of PI for 15 min at room temperature (Solarbio Science & Technology Co., Ltd., Beijing, China). Cell cycle and apoptosis analyses were performed by Calibur flow cytometry (Becton, Dickinson and Company, Franklin Lakes, New Jersey, USA).

### Statistical analysis

R software (version 4.1; PBC, Boston, MA, USA) and GraphPad Prism 7.0 (version 8.0; La Jolla, CA, USA) were used for the statistical analyses. All data are expressed as the mean ± standard deviation. T test and one-way ANOVA were used to compare data between two or more groups that conformed to the normal distribution, respectively. The Wilcoxon rank sum test was used for data that did not conform to a normal distribution, and *p* < 0.05 was considered to indicate a significant difference.

## Results

### Discovery of differentially expressed ARGs

**Figure 2:**
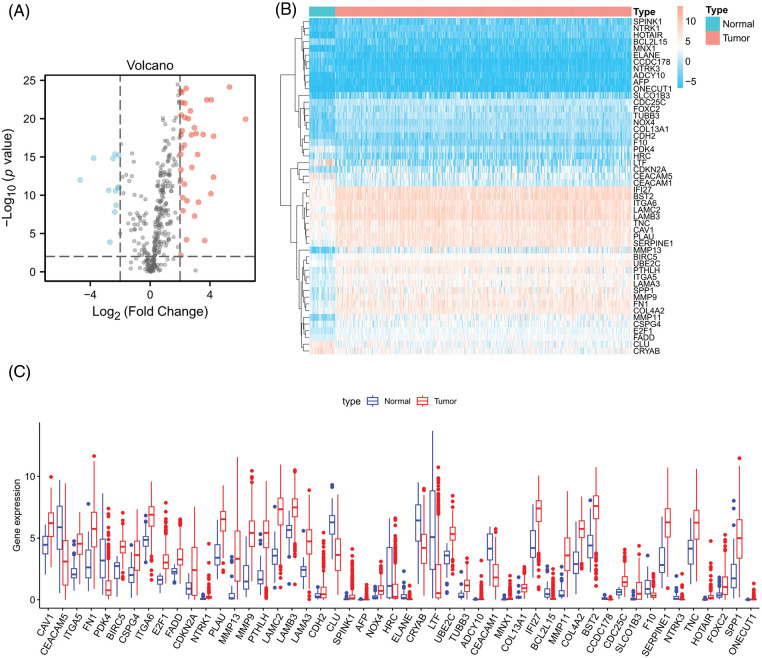
Differentially expressed ARGs. (A) Volcano plot of 364 differentially expressed ARGs. Blue points represent log_2_FC < (−2), and red points represent log_2_FC > 2, **p < 0.01. (B) Heatmap for 50 differentially expressed ARGs. (C) Box plot of differentially expressed ARGs.

Firstly, we downloaded transcript data of 44 normal and 504 cancer samples from the TCGA. Expression values for a total of 364 ARGs were then extracted from HNSCC RNA-seq data (Suppl. Table S2). Fifty differentially expressed ARGs were obtained by screening according to the criteria of *p* < 0.01 and |Log_2_ (Fold Change)| > 2. The expression volcano plot is shown in Fig. 2A. Twelve genes were downregulated in HNSCC tissues compared with the levels in the surrounding normal tissues (LTF, PDK4, ELANE, HRC, BCL2L15, CEACAM5, CEACAM1, NTRK3, CRYAB, CCDC178, CLU, and F10), and 38 genes were upregulated (CAV1, BIRC5, TUBB3, CDH2, NTRK1, LAMB3, FADD, NOX4, CDC25C, UBE2C, ITGA6, CSPG4, FOXC2, IFI27, TNC, COL4A2, COL13A1, E2F1, AFP, MNX1, ITGA5, PLAU, BST2, SPP1, LAMA3, ADCY10, SERPINE1, FN1, SPINK1, PTHLH, CDKN2A, LAMC2, SLCO1B3, MMP9, ONECUT1, HOTAIR, MMP11, and MMP13) (Figs. 2B and 2C).

### Prognostic model establishment and verification

**Figure 3:**
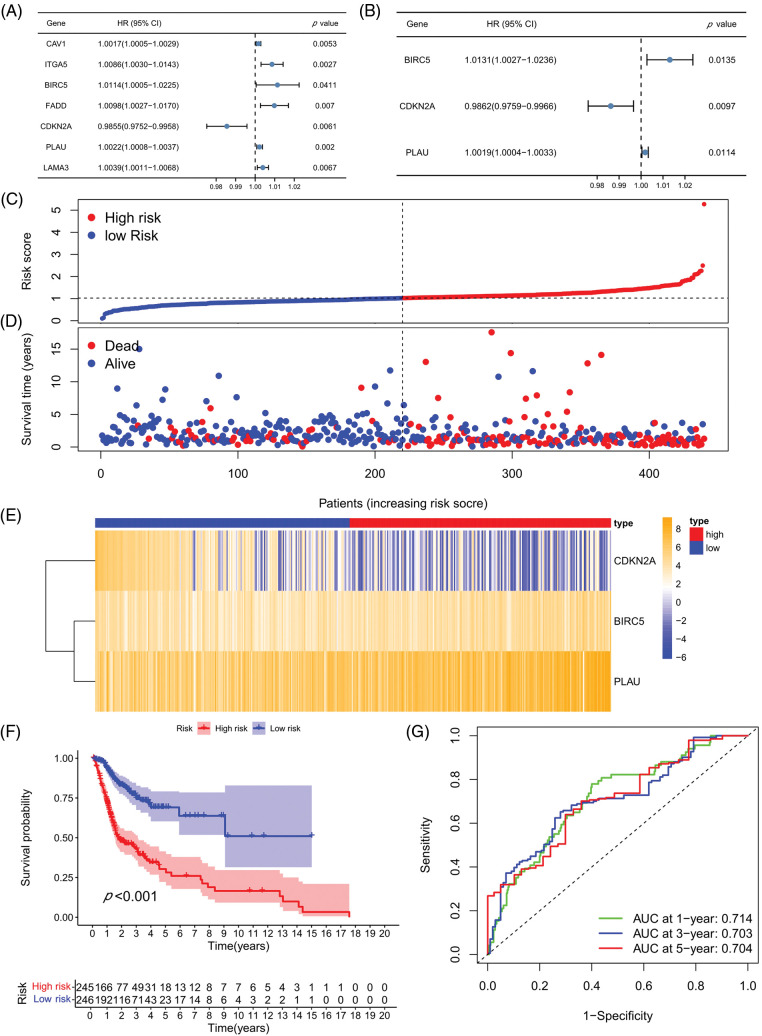
Prognostic model establishment and verification. (A) Univariate COX regression analysis. (B) Multivariate COX regression analysis. (C) Heatmap for the three prognosis-related ARGs. (D) Risk score distribution chart. (E) Distribution of the survival status of the high- and low-risk groups. (F) Kaplan-Meier curves for the prognostic model in high- and low-risk groups. (G) ROC curves and AUC values for 1-, 3-, and 5-year survival. *p < 0.05; **p < 0.01; ***p < 0.001.

A univariate COX analysis was performed on the 50 ARGs, and variables with *p* < 0.05 in the univariate COX analysis were examined (Fig. 3A) in a multivariate analysis. A total of three ARGs (CDKN2A, BIRC5, and PLAU) were significantly associated with prognosis, leading to the construction of a prognostic model (Fig. 3B). We determined risk scores for all cases with expression levels for risk regression coefficients and ARGs. Risk score = CDKN2A expression × (−0.01388) + BIRC5 expression × 0.013027 + PLAU expression × 0.001909. All patients were ranked according to their risk scores, and the differential expression profiles for three ARGs between the high- and low-risk groups were displayed in a heatmap (Fig. 3C), showing that the CDKN2A expression level was obviously decreased in the high-risk group, whereas the BIRC5 and PLAU expression levels were both significantly increased. Further analysis of patient survival status showed that patients in the high-risk group had worse survival outcomes and a higher likelihood of death than those in the low-risk group (Figs. 3D and 3E). The subsequent survival analysis results confirmed that the overall survival (OS) rate of the high-risk group was lower than that of the low-risk group (*p* < 0.001) (Fig. 3F). ROC curves were used to demonstrate the predictive performance of the model, with areas under the curve (AUC) of 0.714, 0.703, and 0.704 for 1-, 3-, and 5-year OS rates, respectively (Fig. 3G), indicating the good predictability of the prognostic model for HNSCC patients.

### Risk scores independently predicted HNSCC prognosis

**Figure 4:**
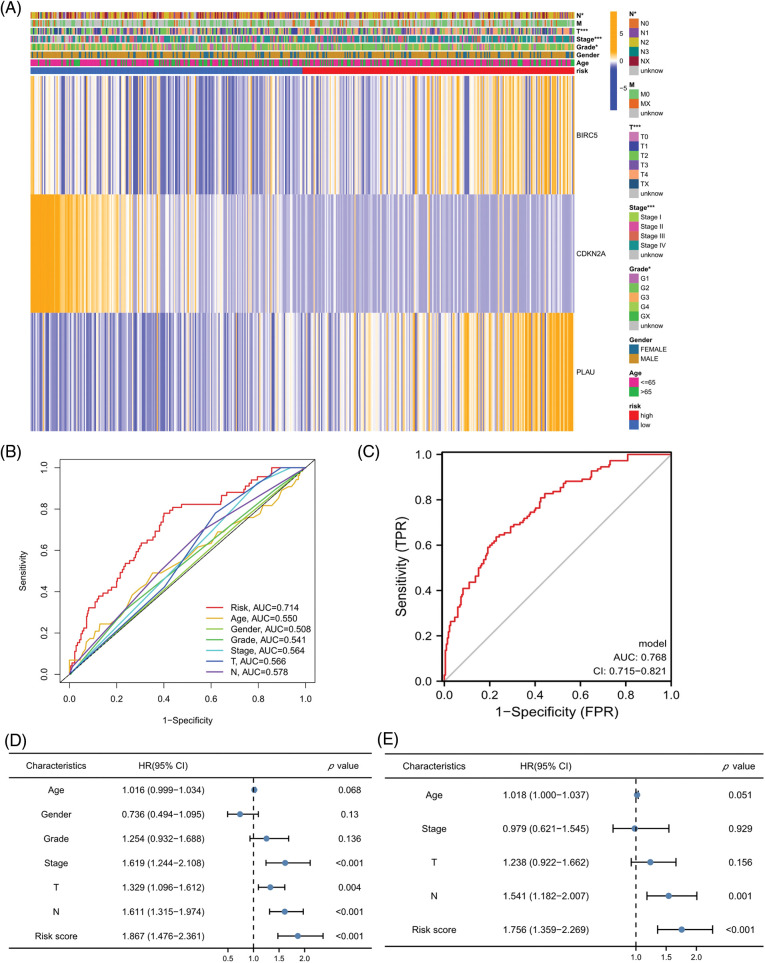
Risk scores independently predict HNSCC prognosis. (A) Heatmap for the correlation between prognosis-related ARGs and clinical indicators. (B) ROC curves and AUC values for risk scores and clinical parameters. (C) Combined ROC curves and AUC values for T and N stage and risk score. (D) Univariate COX regression analysis. (E) Multivariate COX regression analysis. *p < 0.05; **p < 0.01; ***p < 0.001.

The relationship of each clinical parameter to the risk score was investigated by clinicopathological analysis. The results were plotted as a heatmap (Fig. 4A), which showed that the risk scores were significantly associated with N stage (*p* < 0.05), T stage (*p* < 0.001), tumor stage (*p* < 0.001), and grade (*p* < 0.01) but were not relevant to age and sex. The relationship between the risk score and M stage could not be assessed due to the lack of M stage information. The difference in the prognosis predicted by the risk score with other clinicopathological features was assessed by the multivariate ROC curve. As shown in Fig. 4B, the AUC value of the risk score was 0.714, which significantly exceeded that of the additional clinical indicators. The combined ROC curve for T stage, N stage, and risk score was then plotted (Fig. 4C). The AUC value of the combined ROC curve was 0.768, which was significantly higher than that of any other clinical feature. These results suggest that the risk score is a more accurate prognostic predictor for HNSCC patients than other clinical features and a useful complementary means to predict HNSCC patients’ prognosis according to the TNM stage. In addition, we performed univariate and multivariate COX regression analyses to verify the independent prognostic significance of the risk score for HNSCC patient OS outcomes. The univariate COX regression analysis revealed significant differences in age, sex, grade, tumor stage, and risk score (Fig. 4D), while the multivariate COX regression analysis showed that the risk score could be an independent prognostic factor for HNSCC patients (Fig. 4E) (HR = 2.134, 95% CI = 1.524–2.988). In conclusion, the superiority of the model for predicting the prognosis of HNSCC patients was demonstrated. The model-based risk score can be used as an independent prognostic factor in HNSCC patients.

### Investigation of immunity status and clinical treatment response analyses in high- and low-risk groups

**Figure 5:**
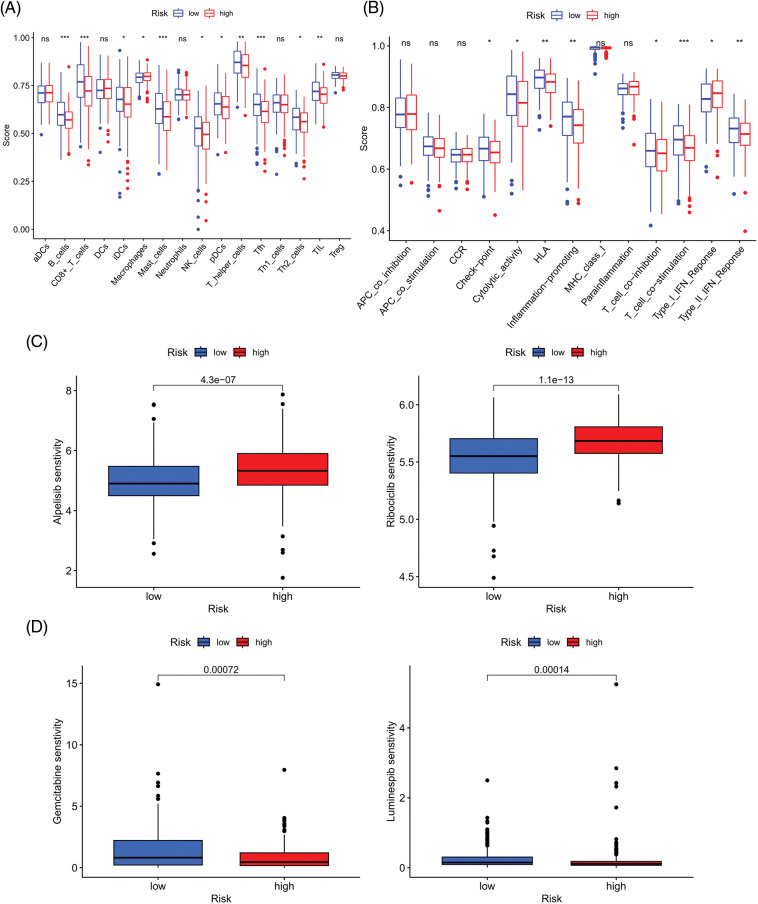
Immune cell infiltration and drug sensitivity analyses in high- and low-risk groups. (A) Comparison of immune cell infiltration in high- and low-risk HNSCC patients. (B) Comparison of immune function between high- and low-risk HNSCC patients. (C) Two representative drugs with lower IC_50_ values in the low-risk group. (D) Two representative drugs with lower IC_50_ values in the high-risk group. *p < 0.05; **p < 0.01; ***p < 0.001.

**FIGURE S1:**
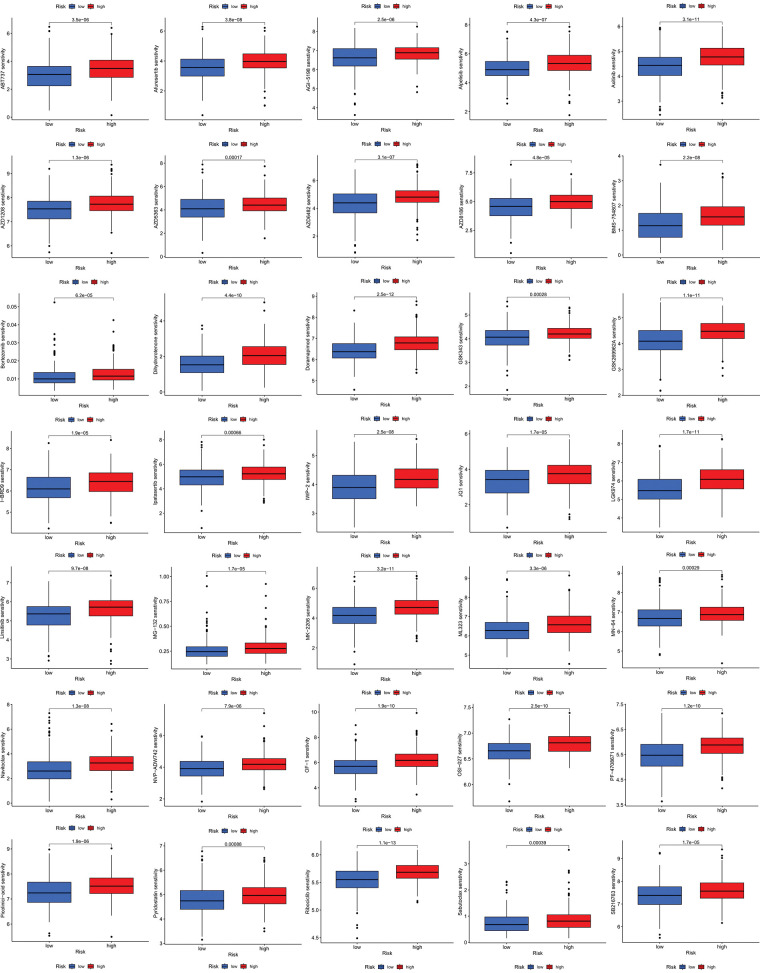

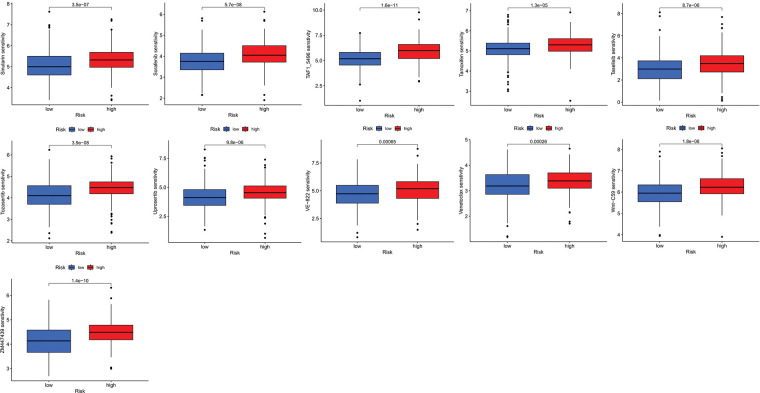
All 41 chemicals with lower IC_50_ values in the low-risk group.

**FIGURE S2:**
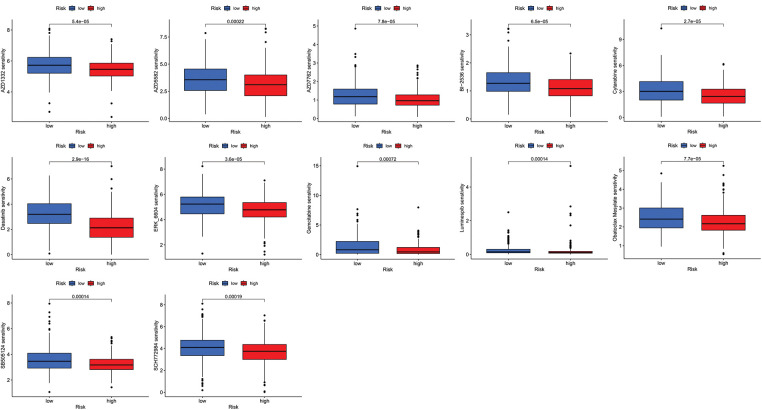
Distribution of CDKN2A, BIRC5, and PLAU expression in different cell types using violin plot, respectively.

We next evaluated the relationship between the risk model and immune infiltration. The immune cell infiltration analysis showed that the low-risk group had higher immune cell infiltration levels, such as B cells, CD8+ T cells, interdigitating dendritic cells (iDCs), macrophages, mast cells, NK cells, plasmacytoid dendritic cells (pDCs), T helper cells, follicular helper T cell (Tfh), T helper 2 cells (Th2), and tumor-infiltrating lymphocytes (TILs) (Fig. 5A). In addition, immune function was compared between the two groups, with the low-risk group demonstrating more prosperous immune function (Fig. 5B). The above results indicate a large difference in immune status between the high- and low-risk groups, with the low-risk group showing higher immune infiltration levels. Sensitivity analysis of 198 drugs was performed in HNSCC. The low-risk group had smaller IC_50_ in 41 drugs, including targeted drugs (e.g., Alpetisib) and immunotherapeutic agents (e.g., Ribociclib) (Fig. 5C), than the high-risk group. All 41 chemicals were shown in Suppl. Fig. S1. Twelve drugs had lower IC_50_ in the high-risk group (Suppl. Fig. S2), of which some drugs including Luminespib were associated with immunotherapy (Fig. 5D).

### External validation of the prognostic model

**Figure 6:**
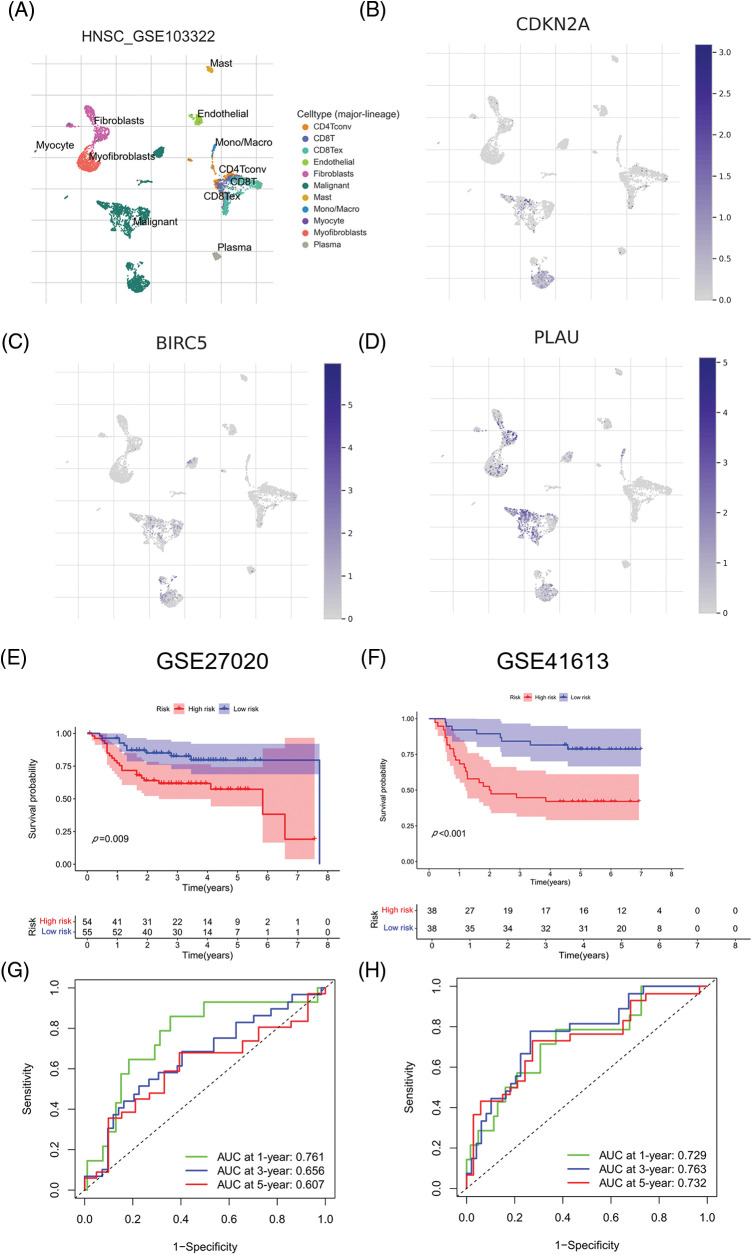
External validation of the three ARGs and prognostic model. (A-D) Distribution of CDKN2A, BIRC5, and PLAU in different cells. (E) Kaplan‒Meier curves for prognostic models of GSE27020 in the high- and low-risk groups. (F) Kaplan‒Meier curves for prognostic models of GSE41613 in the high- and low-risk groups. (G) ROC curves and AUC values for 1-, 3-, and 5-year survival in GSE27020. (H) ROC curves and AUC values for 1-, 3-, and 5-year survival in GSE41613. *p < 0.05; **p < 0.01; ***p < 0.001.

**FIGURE S3:**
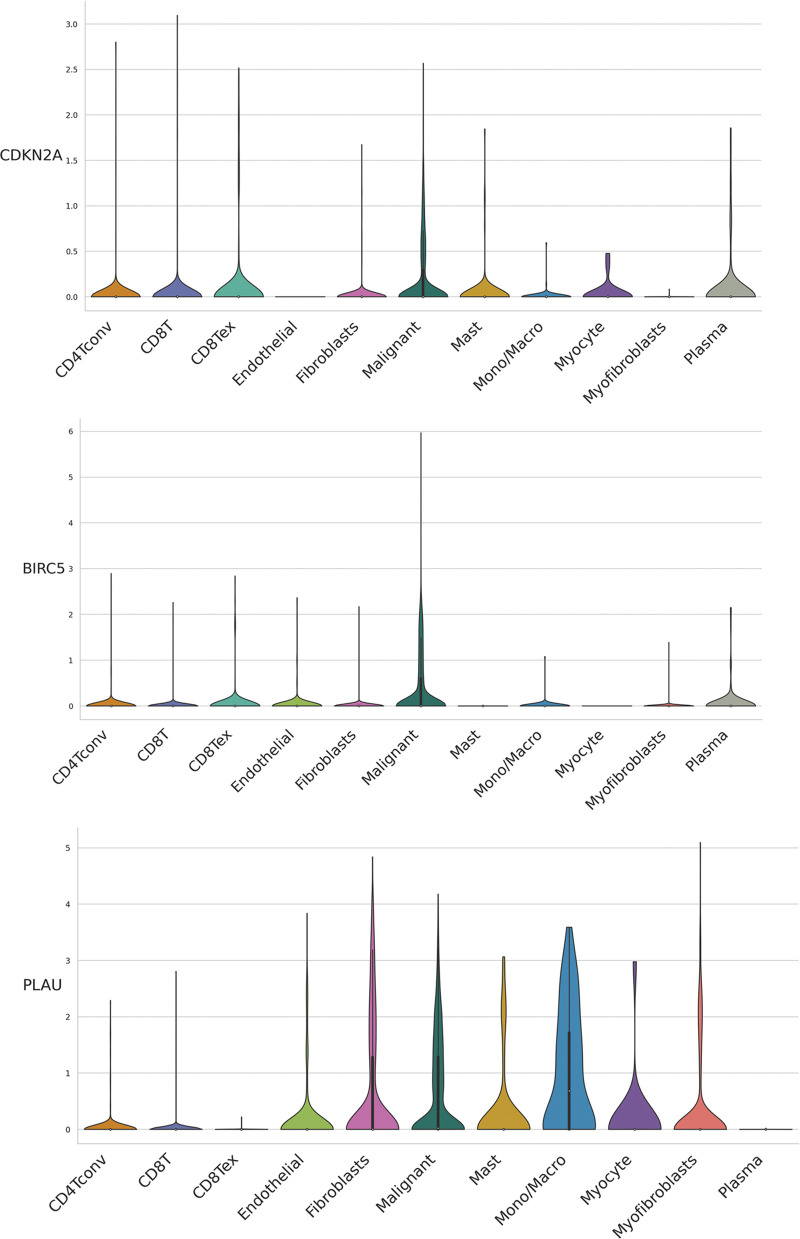
Distribution of CDKN2A, BIRC5, and PLAU expression in different cell types using violin plot, respectively.

We evaluated ARGs expression in HNSCC cells using a dataset from the TISCH database. In the dataset HNSCC_GSE103322, CDKN2A (Figs. 6A and 6B), BIRC5 (Fig. 6C), and PLAU (Fig. 6D) were mainly expressed in HNSCC malignant cells, and their proportions in malignant cells were 76.4%, 50.5%, and 58.9%, respectively (Suppl. Fig. S3). These results were consistent with the results of TCGA database analysis, and again confirmed that CDKN2A, BIRC5, and PLAU have great potential as prognostic markers in HNSCC patients. Then risk scores were calculated for each patient in the GSE27020 and GSE41613 datasets using the same formula used for the external validation of the prognostic model. All patients in the two datasets were divided into high- and low-risk groups as previously described using the median risk score for each dataset. The survival curves of the two datasets were as expected, with significantly lower OS rates in the high-risk group than in the low-risk group (*p* < 0.01) (Figs. 6E and 6F). The ROC curves in GSE27020 had AUC values of 0.719, 0.697, and 0.671 for 1-, 3-, and 5-year OS rates, respectively (Fig. 6G). The AUC values in GSE41613 were 0.729, 0.763, and 0.732 for 1-, 3-, and 5-year OS rates, respectively (Fig. 6H). These results again confirmed the ability of the prognostic model to predict prognosis. Unfortunately, the lack of clinical information, such as sex, T stage, and N stage, prevented the comparison of the predictive ability difference between clinical features and risk scores in the dataset. Nevertheless, the results were sufficient and highlighted the outstanding ability of the model to predict prognosis.

### L1000FWD analysis to identify potential target drugs

**Figure 7:**
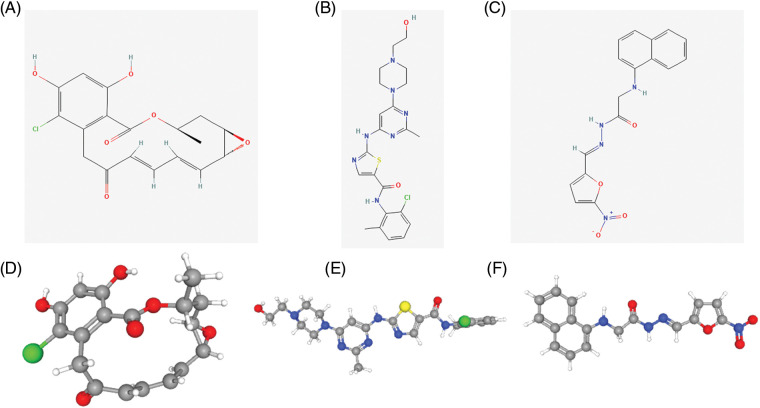
Structure of the top three small molecule drugs. (A) The 2D structure of radicicol. (B) The 2D structure of dasatinib. (C) The 2D structure of BRD-K85660637. (D) The 3D structure of radicicol. (E) The 3D structure of dasatinib. (F) The 3D structure of BRD-K85660637.

We searched for potential target drugs of HNSCC by uploading up- and down-regulated ARGs to the L1000FWD database and obtaining the top ten candidates. The basic drug information is shown in Suppl. Table S3. We selected the top three small-molecule drugs (radicicol, dasatinib, and BRD-K85660637) for visualization. The 2D and 3D structures are shown in Fig. 7. Radicicol, the top-ranked fungicide, was selected for verification in a series of *in vitro* experiments.

### Exploration of the expression characteristics of ARGs and their effect on anoikis in vitro

**Figure 8:**
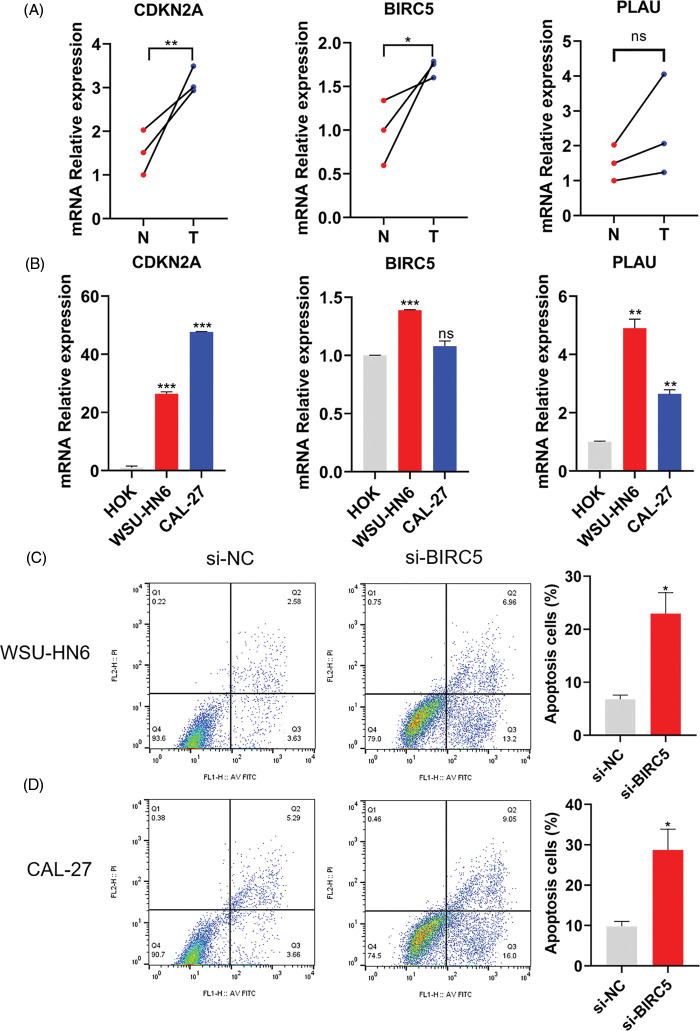
Expression of ARGs in tissues and cell lines and its effect on anoikis. (A) Expression of CDKN2A, BIRC5, and PLAU in three pairs of matched HNSCC (T) and paracancerous normal tissues (N). (B) Expression of CDKN2A, BIRC5, and PLAU in the HOK, WSU-HN6, and CAL-27 cell lines. (C, D) Anoikis of WSU-HN6 and CAL-27 cells and apoptotic cell proportions in each group. The apoptotic cells shown in the graph are the proportion of early anoikis + late anoikis. *p < 0.05, **p < 0.01, ***p < 0.001, compared with si-NC group; n = 3.

The mRNA expression level of three ARGs was detected in three pairs of matched HNSCC (T), paracancerous normal tissues (N), HOK cells, and two HNSCC cell lines. As shown in Figs. 8A and 8B, the relative mRNA expression levels of CDKN2A, BIRC5, and PLAU were higher in HNSCC tissues than in paracancerous normal tissues, while the relative mRNA expression levels of CDKN2A, BIRC5, and PLAU were also higher in both HNSCC cell lines than in HOK cells. Therefore, the expression level of ARGs was consistent with the results of our model analysis. Subsequently, we prevented cell adhesion by cultivating HNSCC cells on nonadherent plates to mimic the environment of cancer cells to verify the correlation between the ARG and anoikis. We selected one of the ARGs (BIRC5) for experimental verification and knocked down BIRC5 in WSU-HN6 and CAL-27 cells, then cultured the cell suspension for 24 h. Flow cytometry was used to assess the proportion of cells undergoing anoikis. The proportion of apoptotic cells was increased significantly in the BIRC5-knockdown group compared with that in the si-NC group (Figs. 8C and 8D). Combined with previous results, the overexpression of ARGs in HNSCC tissues and cells can significantly promote anoikis resistance in tumor cells.

### Radicicol can stably bind to ARGs and inhibit their expression level

**Figure 9:**
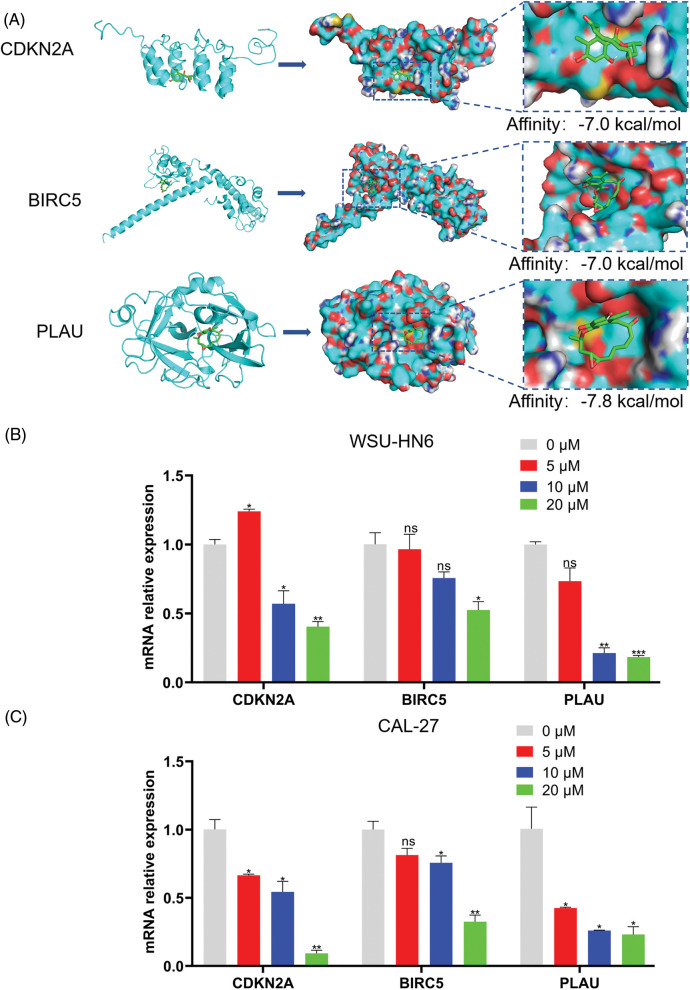
Radicicol may target the ARGs in the prognostic model. (A) Molecular docking diagrams of radicicol with CDKN2A, BIRC5, and PLAU. (B, C) Expression of CDKN2A, BIRC5, and PLAU in the WSU-HN6 and CAL-27 cell lines after coculture with radicicol. *p < 0.05, **p < 0.01, ***p < 0.001, compared with 0 μM; n = 3.

The lower the binding energy between the ligand and the receptor, the more stable it is. Therefore, a binding energy ≤ –5.0 kcal/mol was chosen as the screening condition. In this study, the binding energies of the three ARGs and radicicol were much lower than the set conditions (the binding energies of CDKN2A, BIRC5, and PLAU with radicicol were –7.0, –7.0, and –7.8 kcal/mol, respectively), indicating a good binding effect between radicicol and each ARG (Fig. 9A). We measured the expression levels of CDKN2A, BIRC5, and PLAU to dissect the relationship between radicicol and the three ARGs. RT–PCR showed that the relative mRNA expression levels of CDKN2A, BIRC5, and PLAU were decreased after radicicol treatment (Figs. 9B and 9C). Therefore, radicicol can regulate the mRNA expression level of ARGs, suggesting that radicicol may target the ARGs in the prognostic model.

### Radicicol inhibited the survival rate of HNSCC cells

**Figure 10:**
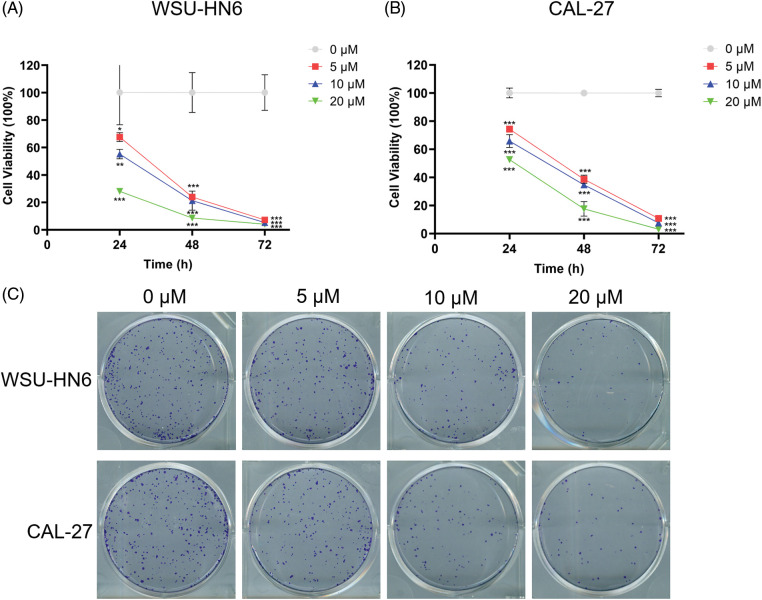
Radicicol inhibited survival rate of WSU-HN6 and CAL-27 cells. (A, B) Survival rate of WSU-HN6 and CAL-27 cells after coculture for 24, 48, and 72 h with radicicol. (C) Plate clone assay to assess the colony formation ability of two cell lines under different concentrations of radicicol. *p < 0.05, **p < 0.01, ***p < 0.001, compared with 0 μM; n = 3.

In the present study, we explored the relationship between the radicicol concentration and the survival rate of HNSCC cells. Three different concentrations (5, 10, and 20 μM) were used to coculture two HNSCC cell lines (WSU-HN6 and CAL-27) for 24, 48, and 72 h. The OD of each well was measured, and the cell viability was subsequently calculated. The results showed that the cell survival rate of both cell lines was significantly reduced with the three different concentrations of radicicol compared with the survival rate in the control group (*p* < 0.05), and radicicol inhibited cells survival rate in a dose- and time-dependent manner (Figs. 10A and 10B). A comparison of the two cell lines revealed that radicicol was more effective in inhibiting the survival rate of CAL-27 cells than WSU-HN6 cells (the mean cell viability rate at each concentration and time point are shown below, and all results are expressed as WSU-HN6 *vs*. CAL-27. 5 μM, 24 h: 79.38% *vs*. 67.63%; 10 μM, 24 h: 69.92% *vs*. 55.20%; 20 μM, 24 h: 69.82% *vs*. 28.03%; 5 μM, 48 h: 48.95% *vs*. 23.93%; 10 μM, 48 h: 39.53% *vs*. 21.28%; 20 μM, 48 h: 27.66% *vs*. 8.59%; 5 μM, 72 h: 37.20% *vs*. 7.25%; 10 μM, 72 h: 29.27% *vs*. 5.29%; 20 μM, 72 h: 20.04% *vs*. 7.13%). Moreover, the number of colonies in the three radicicol groups was reduced compared with that of the control group (Fig. 10C).

### Radicicol can regulate the cell cycle and promote anoikis

**Figure 11:**
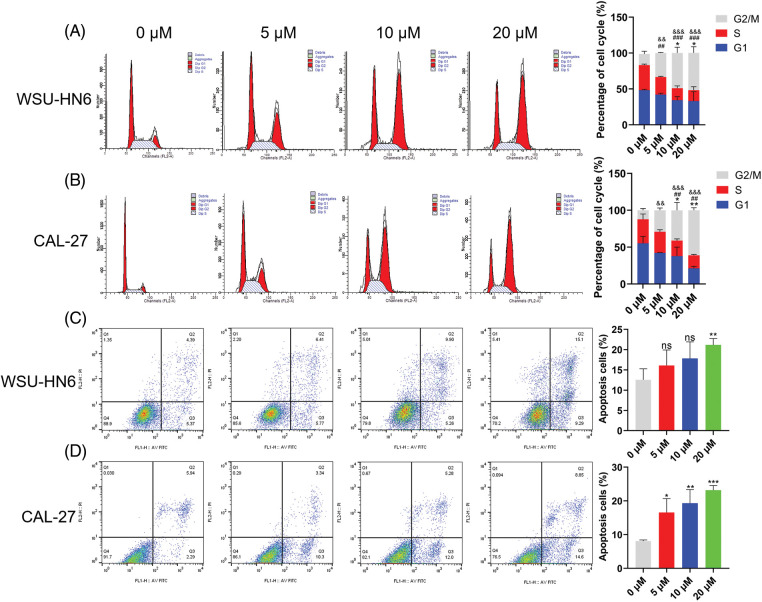
Radicicol can block G_2_/M phase and promote anoikis. (A, B) Distribution and statistics of the cell cycle phases of WSU-HN6 and CAL-27 cells in each group. (C, D) Anoikis of WSU-HN6 and CAL-27 cells and apoptotic cell proportions in each group. The apoptotic cells shown in the graph are the proportion of early anoikis + late anoikis. *^,^^#, &^ *p* < 0.05, **^,^^##, &&^ *p* < 0.01, ***^,^^###, &&&^ *p* < 0.001. * indicates G1 phase or apoptotic cells compared to the radicicol groups at 0 μM, # indicates S phase compared to the radicicol groups at 0 μM; & indicates G2/M phase compared to the radicicol groups at 0 μM; n = 3.

Since cell survival rate was inhibited in the radicicol groups, we hypothesized that one or more cell cycle phases might be blocked during this process. Flow cytometry analysis was performed to test this hypothesis. The radicicol groups had a significantly higher proportion of cells in the G_2_/M phase and a significantly lower proportion in the G1 and S phases than that in the control group (*p* < 0.001) (Figs. 11A and 11B), indicating that the cell cycle was blocked in the G_2_/M phase.

As previously mentioned, HNSCC cells were cultured on nonadherent panels to investigate the effect of radicicol on anoikis. Radicicol was cocultured with suspension culture cells for 24 h, and the cells were stained using Annexin V-FITC/PI to analyze the level of anoikis through the intensity of the cell fluorescence signal. Annexin V-FITC-positive and PI-negative cells represented early anoikis, while Annexin V-FITC-positive and PI-positive cells represented late anoikis. The results showed that the percentage of anoikis cells (percentage of early + late anoikis) was significantly increased in the radicicol groups *vs*. the control group (Figs. 11C and 11D). A dose-dependent effect was demonstrated in all of the above experiments, and the effect of radicicol was more pronounced in CAL-27 cells. These results confirm that radicicol can block the G_2_/M phase and promote anoikis.

## Discussion

The prognosis of HNSCC depends on various factors, including age, lifestyle habits, and treatment, with metastases predominating the prognosis of HNSCC [16]. Several models currently use tumor metastasis to predict the prognosis of HNSCC. TNM staging is currently an accepted model for predicting prognosis; however, N staging only examines the number and size of positive lymph nodes, which are affected by the type of neck lymphatic dissection and the total number of lymph nodes removed [17,18]. The lymph node ratio (LNR) can be a valid predictor of prognosis in HNSCC [19]; however, the LNR cannot be used to assess patient prognosis when the number of positive lymph nodes is zero. Therefore, the creation of a prognostic discriminating model for patients with HNSCC based on tumor metastasis capacity is critical for post-operative patient observation and directing clinical medication use.

Anoikis resistance is a crucial molecular mechanism for survival during the metastatic cascade of tumor cells and is one of the hallmarks of tumorigenesis in EMT and a signature trait of tumor stem cells [20]. The development of anoikis resistance leads to an increased potential for tumor cell metastasis, an expansion of cancer stem cell subpopulations, chemoresistance, and a higher likelihood of recurrence, all of which are significantly related to a poor prognosis in HNSCC [21]. ARG-based predictive models play a significant role in determining the prognosis of many malignancies [22–24]; thus, anoikis resistance has major therapeutic implications. In this study, we used bioinformatics analysis to build an ARG prognostic model for HNSCC to predict patient prognosis and immune activity.

CDKN2A, BIRC5, and PLAU were found to be closely related to patient prognosis, consistent with the results of previous studies. CDKN2A was first identified by Kamb and can encode two proteins, namely, p16INK4a and p14ARF, thus exerting cell cycle regulation through multiple pathways [25]. CDKN2A gene expression abnormalities, primarily deletions, mutations, and abnormal hypermethylation have now been reported in a range of malignancies, including HNSCC [26]. Patients with CDKN2A deletion in HNSCC have a generally poor prognosis and are considerably more likely to experience HNSCC recurrence [27,28]. And targeting CDKN2A and/or the PI3K-AKT-mTOR pathway may be a valuable direction to develop precise therapy for HNSCC [29]. BIRC5 was first isolated and identified in a human gene bank screen by Ambrosini in 1997 and is believed to be the smallest member of the apoptosis suppressor protein family [30]. Moreover, studies have shown that abnormal expression of BIRC5 can be used as a diagnostic marker in HNSCC patients [31] and that BIRC5 is an important predictor of poor prognosis [32]. The PLAU gene is located on human chromosome 10q22.2 and encodes urokinase-type plasminogen activator [33]. PLAU has been shown to be closely related to tumor diagnosis and patient prognosis [34]. Chen demonstrated PLAU may function as an oncogene in HNSCC and regulate the EMT signaling pathway *in vitro* and *in vivo* [35]. Li demonstrated that PI3K-Akt pathway might underly the mechanism of PLAU’s oncogene role in HNSCC [36]. The elevation of CDKN2A, BIRC5, and PLAU expression seen in our tissue samples was consistent with prior studies; however, more clinical samples are needed to determine the link between these three genes’ expression levels and metastasis. In addition, although CDKN2A, BIRC5, and PLAU are associated with the prognosis of HNSCC, their effects on anoikis have not been experimentally verified. We selected BIRC5 to transfect siRNA *in vitro* to detect its effect on anoikis. According to the flow cytometry results, the inhibition of BIRC5 expression increased tumor cell apoptosis and decreased HNSCC anoikis resistance.

According to current studies, the tumor microenvironment (TME) provides a permissive environment for tumor progression and metastasis [37]. Therefore, considering that the TME can regulate anoikis resistance, we conducted ssGSEA to explore the immune status of high- and low-risk groups. The immune cells (B cells, CD8+ T cells, iDCs, macrophages, mast cells, NK cells, pDCs, T helper cells, Tfh, Th2, and TIL) and immune roles (checkpoint, cytolytic activity, HIL, inflammation-promoting, T cell co-inhibition, T cell co-stimulation, and type I (or II) IFN response) were more active in the low-risk group. These results imply that we can use the risk scores of ARGs to effectively distinguish the immune infiltration status of HNSCC and develop personalized immunotherapy. As for drug sensitivity, the low-risk group was more sensitive to multiple inhibitors of the PI3K/AKT/mTOR pathway, such as OSI-027, which not only enhanced immunotherapeutic effects [38] but also blocked the progression of HNSCC [39]. One study has found that PI3K/AKT/mTOR axis is highly activated in HNSCC, which is related to the proliferation, migration, invasion, and other biological behaviors of tumor cells [29]. As a result, our study can guide clinical treatment and provide evidence for improving future immunotherapy and seeking appropriate target populations.

In this study, differentially expressed ARGs were divided into up-regulated and down-regulated groups, with a view to using the database to develop effective drugs against anoikis. The radicicol ranks first among the obtained small molecule drugs. Radicicol, a macrolide antibiotic isolated from *Monosporium bonorden* by Delmotte et al. [40], is still in its infancy as a novel Hsp90 inhibitor. Since Hsp90 has regulatory effects on a variety of substrate proteins, inhibition of Hsp90 can regulate many signaling pathways, thereby inhibiting tumor proliferation, metastasis, and other processes [41], which has made clinically feasible Hsp90 small-molecule inhibitors a research focus. Interestingly, in the previous drug sensitivity analyses, we also found an Hsp90 inhibitor had a lower IC_50_ in the high-risk group, indicating that Hsp90 inhibitor has great potential to become a novel drug targeting ARGs prognostic model. However, radicicol was discovered only a short time ago and has the disadvantage of low activity *in vivo*; thus, it has not yet entered the clinical research stage, especially for HNSCC. A recent study demonstrated that radicicol promoted anoikis in glioblastoma by causing endoplasmic reticulum stress and preventing AKT/mTOR/p3S3K phosphorylation activation, thus confirming that radicicol was closely associated with PI3K/AKT/mTOR and anoikis [42]. In addition, we demonstrated *in vitro* that radicicol modulated mRNA expression levels of the three ARGs included in the prognostic model, and that radicicol inhibited HNSCC cells survival rate and promoted anoikis. In summary, combined with the studies of scholars, we hypothesized that radicicol may promote the anoikis of HNSCC by inhibiting the expressions of CDKN2A, BIRC5, and PLAU, thereby blocking the PI3K/AKT/mTOR signaling pathway. However, the specific molecular mechanism still needs to be confirmed by subsequent experiments.

Our study provides a new perspective for exploring the role of ARGs in the development and metastasis of HNSCC. We constructed a prognostic model, identified novel drug that promotes anoikis, and validated the results of the database analysis *in vitro*. However, it must be acknowledged that there are still some limitations in this study. The model was derived from a public database analysis; thus, its applicability and accuracy need to be further explored in clinical HNSCC patients. The effect of radicicol on anoikis *in vivo* and its molecular mechanisms still need to be further explored. In conclusion, the ARG prognostic model is expected to be a novel biomarker for HNSCC diagnosis and treatment decisions, and ARGs can be considered drug development targets for reducing HNSCC metastasis.

**Supplementary Table 1:** Anoikis-related genes

Gene symbol	Description	Relevance score
BRMS1	BRMS1 Transcriptional Repressor and Anoikis Regulator	14.60019588
PTK2	Protein Tyrosine Kinase 2	7.251347065
NTRK2	Neurotrophic Receptor Tyrosine Kinase 2	7.235249519
BCL2L11	BCL2 Like 11	6.660739422
SRC	SRC Proto-Oncogene, Non-Receptor Tyrosine Kinase	6.154667854
CEACAM6	CEA Cell Adhesion Molecule 6	6.086348057
CAV1	Caveolin 1	5.437981606
AKT1	AKT Serine/Threonine Kinase 1	5.382418633
ITGB1	Integrin Subunit Beta 1	4.980230808
CEACAM5	CEA Cell Adhesion Molecule 5	4.643890381
EGFR	Epidermal Growth Factor Receptor	4.588373184
BCL2	BCL2 Apoptosis Regulator	4.521504879
CASP8	Caspase 8	4.462318897
PTRH2	Peptidyl-TRNA Hydrolase 2	4.177913189
STAT3	Signal Transducer and Activator of Transcription 3	4.116282463
SIK1	Salt Inducible Kinase 1	4.061151028
TLE1	TLE Family Member 1, Transcriptional Corepressor	4.055632114
DAPK2	Death Associated Protein Kinase 2	3.980505228
CTNNB1	Catenin Beta 1	3.962141991
ZNF304	Zinc Finger Protein 304	3.932430744
BMF	Bcl2 Modifying Factor	3.717435837
MAPK1	Mitogen-Activated Protein Kinase 1	3.712444782
ITGA5	Integrin Subunit Alpha 5	3.662759542
MCL1	MCL1 Apoptosis Regulator, BCL2 Family Member	3.564844131
TP53	Tumor Protein P53	3.476554871
BCL2L1	BCL2 Like 1	3.349056244
CASP3	Caspase 3	3.101522207
CDH1	Cadherin 1	3.041518211
BAD	BCL2 Associated Agonist of Cell Death	2.945515156
PIK3CA	Phosphatidylinositol-4,5-Bisphosphate 3-Kinase Catalytic Subunit Alpha	2.934398174
PAK1	P21 (RAC1) Activated Kinase 1	2.913595915
ITGAV	Integrin Subunit Alpha V	2.858327866
FN1	Fibronectin 1	2.804813862
MAPK3	Mitogen-Activated Protein Kinase 3	2.713708639
PTGS2	Prostaglandin-Endoperoxide Synthase 2	2.676221848
BAX	BCL2 Associated X, Apoptosis Regulator	2.533073187
BCAR1	BCAR1 Scaffold Protein, Cas Family Member	2.521586895
PTEN	Phosphatase and Tensin Homolog	2.503646851
ERBB2	Erb-B2 Receptor Tyrosine Kinase 2	2.419464111
PDK4	Pyruvate Dehydrogenase Kinase 4	2.416656733
ANGPTL4	Angiopoietin Like 4	2.384259224
CYCS	Cytochrome C, Somatic	2.325665951
ANKRD13C	Ankyrin Repeat Domain 13C	2.324474096
BRAF	B-Raf Proto-Oncogene, Serine/Threonine Kinase	2.321515799
YAP1	Yes1 Associated Transcriptional Regulator	2.318353176
ANXA5	Annexin A5	2.2526052
MTOR	Mechanistic Target of Rapamycin Kinase	2.243305206
BIRC5	Baculoviral IAP Repeat Containing 5	2.242398739
TIMP1	TIMP Metallopeptidase Inhibitor 1	2.232094526
BDNF	Brain Derived Neurotrophic Factor	2.208322048
ITGA2	Integrin Subunit Alpha 2	2.208115101
BSG	Basigin (Ok Blood Group)	2.182199478
CSPG4	Chondroitin Sulfate Proteoglycan 4	2.182199478
AKT2	AKT Serine/Threonine Kinase 2	2.169704676
STK11	Serine/Threonine Kinase 11	2.144534826
IGF1	Insulin Like Growth Factor 1	2.13505435
IGF1R	Insulin Like Growth Factor 1 Receptor	2.134195566
ITGA6	Integrin Subunit Alpha 6	2.09735322
ILK	Integrin Linked Kinase	2.070893526
CFLAR	CASP8 and FADD Like Apoptosis Regulator	2.070689201
RHOA	Ras Homolog Family Member A	2.056363583
HIF1A	Hypoxia Inducible Factor 1 Subunit Alpha	2.05274415
DAP3	Death Associated Protein 3	2.04320097
MYBBP1A	MYB Binding Protein 1a	2.015814781
TLE5	TLE Family Member 5, Transcriptional Modulator	1.993301392
ITGA3	Integrin Subunit Alpha 3	1.986256361
PTK2B	Protein Tyrosine Kinase 2 Beta	1.983302951
CCND1	Cyclin D1	1.969666362
CTTN	Cortactin	1.969666362
CALR	Calreticulin	1.933260679
CDCP1	CUB Domain Containing Protein 1	1.920459867
CHEK2	Checkpoint Kinase 2	1.902872562
SKP2	S-Phase Kinase Associated Protein 2	1.896605611
E2F1	E2F Transcription Factor 1	1.875796556
HGF	Hepatocyte Growth Factor	1.872206092
EGF	Epidermal Growth Factor	1.859656096
PIK3CG	Phosphatidylinositol-4,5-Bisphosphate 3-Kinase Catalytic Subunit Gamma	1.856436491
ITGB4	Integrin Subunit Beta 4	1.844677448
DAPK1	Death Associated Protein Kinase 1	1.834669709
PIK3R1	Phosphoinositide-3-Kinase Regulatory Subunit 1	1.809942007
MAP2K1	Mitogen-Activated Protein Kinase Kinase 1	1.785508752
CXCL12	C-X-C Motif Chemokine Ligand 12	1.766370296
LGALS3	Galectin 3	1.73115778
BAK1	BCL2 Antagonist/Killer 1	1.719797611
ABHD4	Abhydrolase Domain Containing 4, N-Acyl Phospholipase B	1.696367621
CD44	CD44 Molecule (Indian Blood Group)	1.692866564
FADD	Fas Associated via Death Domain	1.679706693
ITGA4	Integrin Subunit Alpha 4	1.679706693
HMCN1	Hemicentin 1	1.675498486
TGFB1	Transforming Growth Factor Beta 1	1.675498486
CEBPB	CCAAT Enhancer Binding Protein Beta	1.662439466
MMP2	Matrix Metallopeptidase 2	1.662439466
CDKN3	Cyclin Dependent Kinase Inhibitor 3	1.656830788
CASP9	Caspase 9	1.644494295
CBL	Cbl Proto-Oncogene	1.644494295
MTDH	Metadherin	1.644494295
SFN	Stratifin	1.644494295
CXCL8	C-X-C Motif Chemokine Ligand 8	1.625784397
TNFRSF10B	TNF Receptor Superfamily Member 10b	1.625784397
AR	Androgen Receptor	1.608912826
CDKN2A	Cyclin Dependent Kinase Inhibitor 2A	1.60620296
CLDN1	Claudin 1	1.60620296
CPT1A	Carnitine Palmitoyltransferase 1A	1.60620296
MAPK8	Mitogen-Activated Protein Kinase 8	1.60620296
PIK3CB	Phosphatidylinositol-4,5-Bisphosphate 3-Kinase Catalytic Subunit Beta	1.60620296
ATF4	Activating Transcription Factor 4	1.585615396
CDKN1A	Cyclin Dependent Kinase Inhibitor 1A	1.585615396
CDKN1B	Cyclin Dependent Kinase Inhibitor 1B	1.585615396
KLF12	Kruppel Like Factor 12	1.585615396
NTRK1	Neurotrophic Receptor Tyrosine Kinase 1	1.564219713
LGALS1	Galectin 1	1.563848495
MUC1	Mucin 1, Cell Surface Associated	1.563848495
MYC	MYC Proto-Oncogene, BHLH Transcription Factor	1.563848495
PLAU	Plasminogen Activator, Urokinase	1.563848495
PLAUR	Plasminogen Activator, Urokinase Receptor	1.563848495
PLK1	Polo Like Kinase 1	1.563848495
PYCARD	PYD and CARD Domain Containing	1.563848495
SESN2	Sestrin 2	1.563848495
SMAD4	SMAD Family Member 4	1.563848495
ITGB3	Integrin Subunit Beta 3	1.559146404
KRAS	KRAS Proto-Oncogene, GTPase	1.559146404
BID	BH3 Interacting Domain Death Agonist	1.540673137
THBS1	Thrombospondin 1	1.540673137
HRAS	HRas Proto-Oncogene, GTPase	1.525928378
CDK11A	Cyclin Dependent Kinase 11A	1.515774131
CDK11B	Cyclin Dependent Kinase 11B	1.515774131
XIAP	X-Linked Inhibitor of Apoptosis	1.509104013
IL6	Interleukin 6	1.488698006
PPARG	Peroxisome Proliferator Activated Receptor Gamma	1.488698006
CCR7	C-C Motif Chemokine Receptor 7	1.458749056
MSLN	Mesothelin	1.458749056
GRHL2	Grainyhead Like Transcription Factor 2	1.452867508
RAC1	Rac Family Small GTPase 1	1.452867508
NOTCH1	Notch Receptor 1	1.436406255
CCAR2	Cell Cycle and Apoptosis Regulator 2	1.427730918
RHOG	Ras Homolog Family Member G	1.427730918
NQO1	NAD(P)H Quinone Dehydrogenase 1	1.424755216
BIRC3	Baculoviral IAP Repeat Containing 3	1.422440529
MMP13	Matrix Metallopeptidase 13	1.39032352
FAS	Fas Cell Surface Death Receptor	1.387337685
MTA1	Metastasis Associated 1	1.387337685
CCN6	Cellular Communication Network Factor 6	1.384432435
EDA2R	Ectodysplasin A2 Receptor	1.384432435
MYO5A	Myosin VA	1.384432435
ABL1	ABL Proto-Oncogene 1, Non-Receptor Tyrosine Kinase	1.366204023
MAPK11	Mitogen-Activated Protein Kinase 11	1.366204023
MMP9	Matrix Metallopeptidase 9	1.366204023
PTHLH	Parathyroid Hormone Like Hormone	1.362438798
GLI2	GLI Family Zinc Finger 2	1.34561646
PDGFB	Platelet Derived Growth Factor Subunit B	1.34561646
EZH2	Enhancer Of Zeste 2 Polycomb Repressive Complex 2 Subunit	1.344480634
CXCR4	C-X-C Motif Chemokine Receptor 4	1.335362673
RIPK1	Receptor Interacting Serine/Threonine Kinase 1	1.326765537
HMGA1	High Mobility Group AT-Hook 1	1.323849559
SIK2	Salt Inducible Kinase 2	1.323849559
TNFSF10	TNF Superfamily Member 10	1.323849559
ANGPTL2	Angiopoietin Like 2	1.305413604
ETV4	ETS Variant Transcription Factor 4	1.3006742
NTF3	Neurotrophin 3	1.3006742
S100A4	S100 Calcium Binding Protein A4	1.3006742
CEACAM3	CEA Cell Adhesion Molecule 3	1.275775194
HTRA1	HtrA Serine Peptidase 1	1.275775194
LATS1	Large Tumor Suppressor Kinase 1	1.275775194
EIF2AK3	Eukaryotic Translation Initiation Factor 2 Alpha Kinase 3	1.272843242
LAMA3	Laminin Subunit Alpha 3	1.272843242
LAMB3	Laminin Subunit Beta 3	1.272843242
LAMC2	Laminin Subunit Gamma 2	1.272843242
CDH2	Cadherin 2	1.253261805
CSNK2A1	Casein Kinase 2 Alpha 1	1.253261805
EDIL3	EGF Like Repeats and Discoidin Domains 3	1.253261805
TLN1	Talin 1	1.248699188
ZEB2	Zinc Finger E-Box Binding Homeobox 2	1.248699188
CEMIP	Cell Migration Inducing Hyaluronidase 1	1.232674241
EPHA2	EPH Receptor A2	1.232674241
OLFM3	Olfactomedin 3	1.232674241
SIRT3	Sirtuin 3	1.232674241
SOD2	Superoxide Dismutase 2	1.232674241
CLU	Clusterin	1.21875
CPEB2	Cytoplasmic Polyadenylation Element Binding Protein 2	1.21875
SPINK1	Serine Peptidase Inhibitor Kazal Type 1	1.21875
NAT1	N-Acetyltransferase 1	1.21090734
TSG101	Tumor Susceptibility 101	1.21090734
SERPINA1	Serpin Family A Member 1	1.205016255
AFP	Alpha Fetoprotein	1.187731981
AKT3	AKT Serine/Threonine Kinase 3	1.187731981
CD63	CD63 Molecule	1.187731981
EEF1A1	Eukaryotic Translation Elongation Factor 1 Alpha 1	1.187731981
FASLG	Fas Ligand	1.187731981
HRC	Histidine Rich Calcium Binding Protein	1.187731981
ITGA8	Integrin Subunit Alpha 8	1.187731981
LTB4R2	Leukotriene B4 Receptor 2	1.187731981
MAVS	Mitochondrial Antiviral Signaling Protein	1.187731981
NOX4	NADPH Oxidase 4	1.187731981
PBK	PDZ Binding Kinase	1.187731981
PRKCA	Protein Kinase C Alpha	1.187731981
RELA	RELA Proto-Oncogene, NF-KB Subunit	1.187731981
SATB1	SATB Homeobox 1	1.187731981
TNFRSF1A	TNF Receptor Superfamily Member 1A	1.187731981
CCN2	Cellular Communication Network Factor 2	1.184756279
PPP1R13B	Protein Phosphatase 1 Regulatory Subunit 13B	1.184756279
RHOB	Ras Homolog Family Member B	1.184756279
PLG	Plasminogen	1.178547263
MET	MET Proto-Oncogene, Receptor Tyrosine Kinase	1.177064657
BRCA2	BRCA2 DNA Repair Associated	1.162832975
CD24	CD24 Molecule	1.162832975
DOCK1	Dedicator of Cytokinesis 1	1.162832975
HAVCR2	Hepatitis A Virus Cellular Receptor 2	1.162832975
INHBB	Inhibin Subunit Beta B	1.162832975
PARP1	Poly(ADP-Ribose) Polymerase 1	1.162832975
PDCD4	Programmed Cell Death 4	1.162832975
PHLDA2	Pleckstrin Homology Like Domain Family A Member 2	1.162832975
PRKCQ	Protein Kinase C Theta	1.162832975
PRPF4B	Pre-MRNA Processing Factor 4B	1.162832975
RAF1	Raf-1 Proto-Oncogene, Serine/Threonine Kinase	1.162832975
RANBP9	RAN Binding Protein 9	1.162832975
RB1	RB Transcriptional Corepressor 1	1.162832975
SESN1	Sestrin 1	1.162832975
SESN3	Sestrin 3	1.162832975
SP1	Sp1 Transcription Factor	1.162832975
VTN	Vitronectin	1.162832975
ZBTB7A	Zinc Finger and BTB Domain Containing 7A	1.162832975
ELANE	Elastase, Neutrophil Expressed	1.144433498
ABHD2	Abhydrolase Domain Containing 2, Acylglycerol Lipase	1.135756969
CRYAB	Crystallin Alpha B	1.135756969
EPHB6	EPH Receptor B6	1.135756969
FGF2	Fibroblast Growth Factor 2	1.135756969
HK2	Hexokinase 2	1.135756969
IQGAP1	IQ Motif Containing GTPase Activating Protein 1	1.135756969
KDR	Kinase Insert Domain Receptor	1.135756969
KL	Klotho	1.135756969
LTF	Lactotransferrin	1.135756969
MDM2	MDM2 Proto-Oncogene	1.135756969
MGAT5	Alpha-1,6-Mannosylglycoprotein6-Beta-N Acetylglucosaminyltransferase	1.135756969
NFE2L2	NFE2 Like BZIP Transcription Factor 2	1.135756969
PRKCI	Protein Kinase C Iota	1.135756969
SDCBP	Syndecan Binding Protein	1.135756969
SPIB	Spi-B Transcription Factor	1.135756969
TRIM31	Tripartite Motif Containing 31	1.135756969
ZEB1	Zinc Finger E-Box Binding Homeobox 1	1.135756969
BMP6	Bone Morphogenetic Protein 6	1.105807781
BNIP3	BCL2 Interacting Protein 3	1.105807781
BNIP3L	BCL2 Interacting Protein 3 Like	1.105807781
CASP10	Caspase 10	1.105807781
ELK1	ETS Transcription Factor ELK1	1.105807781
KDM3A	Lysine Demethylase 3A	1.105807781
LMO3	LIM Domain Only 3	1.105807781
NRAS	NRAS Proto-Oncogene, GTPase	1.105807781
PAK4	P21 (RAC1) Activated Kinase 4	1.105807781
PDGFRB	Platelet Derived Growth Factor Receptor Beta	1.105807781
PIN1	Peptidylprolyl Cis/Trans Isomerase, NIMA-Interacting 1	1.105807781
PLAT	Plasminogen Activator, Tissue Type	1.105807781
PRDX4	Peroxiredoxin 4	1.105807781
ROCK1	Rho Associated Coiled-Coil Containing Protein Kinase 1	1.105807781
TLR3	Toll Like Receptor 3	1.105807781
TWIST1	Twist Family BHLH Transcription Factor 1	1.105807781
UBE2C	Ubiquitin Conjugating Enzyme E2 C	1.105807781
VEGFA	Vascular Endothelial Growth Factor A	1.105807781
YWHAZ	Tyrosine 3-Monooxygenase/Tryptophan 5-Monooxygenase Activation Protein Zeta	1.105807781
ZNF32	Zinc Finger Protein 32	1.105807781
TUBB3	Tubulin Beta 3 Class III	1.091883659
ADCY10	Adenylate Cyclase 10	1.07181406
APOBEC3G	Apolipoprotein B MRNA Editing Enzyme Catalytic Subunit 3G	1.07181406
BAG1	BAG Cochaperone 1	1.07181406
BCL2L15	BCL2 Like 15	1.07181406
CASP6	Caspase 6	1.07181406
CD36	CD36 Molecule	1.07181406
CDH3	Cadherin 3	1.07181406
CEACAM1	CEA Cell Adhesion Molecule 1	1.07181406
COL13A1	Collagen Type XIII Alpha 1 Chain	1.07181406
EEF2K	Eukaryotic Elongation Factor 2 Kinase	1.07181406
GDF2	Growth Differentiation Factor 2	1.07181406
GLO1	Glyoxalase I	1.07181406
HMOX1	Heme Oxygenase 1	1.07181406
IFI27	Interferon Alpha Inducible Protein 27	1.07181406
IL17A	Interleukin 17A	1.07181406
ITPRIP	Inositol 1,4,5-Trisphosphate Receptor Interacting Protein	1.07181406
LPAR1	Lysophosphatidic Acid Receptor 1	1.07181406
LRP1	LDL Receptor Related Protein 1	1.07181406
MNX1	Motor Neuron and Pancreas Homeobox 1	1.07181406
PAK2	P21 (RAC1) Activated Kinase 2	1.07181406
PAK3	P21 (RAC1) Activated Kinase 3	1.07181406
PIK3R2	Phosphoinositide-3-Kinase Regulatory Subunit 2	1.07181406
PIK3R3	Phosphoinositide-3-Kinase Regulatory Subunit 3	1.07181406
PPP2CA	Protein Phosphatase 2 Catalytic Subunit Alpha	1.07181406
PRKACA	Protein Kinase CAMP-Activated Catalytic Subunit Alpha	1.07181406
PTK6	Protein Tyrosine Kinase 6	1.07181406
PTPN11	Protein Tyrosine Phosphatase Non-Receptor Type 11	1.07181406
RAD9A	RAD9 Checkpoint Clamp Component A	1.07181406
RBL2	RB Transcriptional Corepressor Like 2	1.07181406
SIRPA	Signal Regulatory Protein Alpha	1.07181406
SLC2A1	Solute Carrier Family 2 Member 1	1.07181406
TNFRSF12A	TNF Receptor Superfamily Member 12A	1.07181406
TRAF2	TNF Receptor Associated Factor 2	1.07181406
VPS37A	VPS37A Subunit Of ESCRT-I	1.07181406
SNAI2	Snail Family Transcriptional Repressor 2	1.050797462
ARHGEF7	Rho Guanine Nucleotide Exchange Factor 7	1.03149128
BST2	Bone Marrow Stromal Cell Antigen 2	1.03149128
CCDC178	Coiled-Coil Domain Containing 178	1.03149128
CCN1	Cellular Communication Network Factor 1	1.03149128
CD151	CD151 Molecule (Raph Blood Group)	1.03149128
COL4A2	Collagen Type IV Alpha 2 Chain	1.03149128
CTNND1	Catenin Delta 1	1.03149128
FASN	Fatty Acid Synthase	1.03149128
GLUD1	Glutamate Dehydrogenase 1	1.03149128
MMP11	Matrix Metallopeptidase 11	1.03149128
MYH9	Myosin Heavy Chain 9	1.03149128
NOTCH3	Notch Receptor 3	1.03149128
PPP2R1A	Protein Phosphatase 2 Scaffold Subunit Aalpha	1.03149128
PPP2R2D	Protein Phosphatase 2 Regulatory Subunit Bdelta	1.03149128
PPP2R5A	Protein Phosphatase 2 Regulatory Subunit B’Alpha	1.03149128
PTPN1	Protein Tyrosine Phosphatase Non-Receptor Type 1	1.03149128
RPS6KB1	Ribosomal Protein S6 Kinase B1	1.03149128
SIRT1	Sirtuin 1	1.03149128
TPM1	Tropomyosin 1	1.03149128
SHC1	SHC Adaptor Protein 1	1.010520816
BCL2L2	BCL2 Like 2	0.978941441
BUB1	BUB1 Mitotic Checkpoint Serine/Threonine Kinase	0.978941441
BUB3	BUB3 Mitotic Checkpoint Protein	0.978941441
CDC25C	Cell Division Cycle 25C	0.978941441
CDK1	Cyclin Dependent Kinase 1	0.978941441
DLG1	Discs Large MAGUK Scaffold Protein 1	0.978941441
DYNLL2	Dynein Light Chain LC8-Type 2	0.978941441
EDAR	Ectodysplasin A Receptor	0.978941441
FER	FER Tyrosine Kinase	0.978941441
ITGB5	Integrin Subunit Beta 5	0.978941441
MAD2L1	Mitotic Arrest Deficient 2 Like 1	0.978941441
PDCD6IP	Programmed Cell Death 6 Interacting Protein	0.978941441
SCRIB	Scribble Planar Cell Polarity Protein	0.978941441
SETD2	SET Domain Containing 2, Histone Lysine Methyltransferase	0.978941441
SH3GLB1	SH3 Domain Containing GRB2 Like, Endophilin B1	0.978941441
SLCO1B3	Solute Carrier Organic Anion Transporter Family Member 1B3	0.978941441
TDGF1	Teratocarcinoma-Derived Growth Factor 1	0.978941441
TP73	Tumor Protein P73	0.978941441
TSC2	TSC Complex Subunit 2	0.959778547
MAP3K7	Mitogen-Activated Protein Kinase Kinase Kinase 7	0.919455707
BAG4	BAG Cochaperone 4	0.915065408
ADAMTSL1	ADAMTS Like 1	0.8520751
F10	Coagulation Factor X	0.8520751
F3	Coagulation Factor III, Tissue Factor	0.8520751
HSP90B1	Heat Shock Protein 90 Beta Family Member 1	0.8520751
SERPINB1	Serpin Family B Member 1	0.8520751
CTBP1	C-Terminal Binding Protein 1	0.833141267
MAP3K1	Mitogen-Activated Protein Kinase Kinase Kinase 1	0.833141267
CEACAM4	CEA Cell Adhesion Molecule 4	0.799147487
PXN	Paxillin	0.786795497
MALAT1	Metastasis Associated Lung Adenocarcinoma Transcript 1	0.783030272
CRYBA1	Crystallin Beta A1	0.740039587
IKBKG	Inhibitor of Nuclear Factor Kappa B Kinase Regulatory Subunit Gamma	0.740039587
TFDP1	Transcription Factor Dp-1	0.740039587
SERPINE1	Serpin Family E Member 1	0.726005077
FOXO3	Forkhead Box O3	0.724749267
ACTG1	Actin Gamma 1	0.706274867
ARHGDIA	Rho GDP Dissociation Inhibitor Alpha	0.706274867
EZR	Ezrin	0.706274867
SLC39A6	Solute Carrier Family 39 Member 6	0.706274867
BIN1	Bridging Integrator 1	0.696366668
TIAM1	TIAM Rac1 Associated GEF 1	0.696366668
PDPK1	3-Phosphoinositide Dependent Protein Kinase 1	0.692011356
SMAD7	SMAD Family Member 7	0.669290602
NTRK3	Neurotrophic Receptor Tyrosine Kinase 3	0.639341533
RHOC	Ras Homolog Family Member C	0.639341533
CASP2	Caspase 2	0.631498814
IRF6	Interferon Regulatory Factor 6	0.608323455
TNC	Tenascin C	0.608323455
HOTAIR	HOX Transcript Antisense RNA	0.605347753
GNE	Glucosamine (UDP-N-Acetyl)-2-Epimerase/N-Acetylmannosamine Kinase	0.583424449
XAF1	XIAP Associated Factor 1	0.583424449
SFRP1	Secreted Frizzled Related Protein 1	0.579408526
ARHGDIB	Rho GDP Dissociation Inhibitor Beta	0.565024912
CCDC80	Coiled-Coil Domain Containing 80	0.565024912
CSK	C-Terminal Src Kinase	0.565024912
ENDOG	Endonuclease G	0.565024912
FBLIM1	Filamin Binding LIM Protein 1	0.565024912
FOXC2	Forkhead Box C2	0.565024912
MAP2K2	Mitogen-Activated Protein Kinase Kinase 2	0.565024912
PIK3C2B	Phosphatidylinositol-4-Phosphate 3-Kinase Catalytic Subunit Type 2 Beta	0.565024912
RACK1	Receptor For Activated C Kinase 1	0.565024912
TAGLN	Transgelin	0.565024912
PRKD1	Protein Kinase D1	0.556348383
ANXA2	Annexin A2	0.526399314
LDHA	Lactate Dehydrogenase A	0.526399314
QSOX1	Quiescin Sulfhydryl Oxidase 1	0.526399314
RBFOX2	RNA Binding Fox-1 Homolog 2	0.526399314
SMARCE1	SWI/SNF Related, Matrix Associated, Actin Dependent Regulator Of Chromatin, Subfamily E, Member 1	0.526399314
SPP1	Secreted Phosphoprotein 1	0.526399314
AFAP1L1	Actin Filament Associated Protein 1 Like 1	0.492405564
ATF2	Activating Transcription Factor 2	0.492405564
CDC42	Cell Division Cycle 42	0.492405564
CEACAM8	CEA Cell Adhesion Molecule 8	0.492405564
CRABP2	Cellular Retinoic Acid Binding Protein 2	0.492405564
ID2	Inhibitor of DNA Binding 2	0.492405564
JUP	Junction Plakoglobin	0.492405564
MAOA	Monoamine Oxidase A	0.492405564
NKX2-1	NK2 Homeobox 1	0.492405564
OCLN	Occludin	0.492405564
PIP5K1C	Phosphatidylinositol-4-Phosphate 5-Kinase Type 1 Gamma	0.492405564
PITPNC1	Phosphatidylinositol Transfer Protein Cytoplasmic 1	0.492405564
RPS6KA3	Ribosomal Protein S6 Kinase A3	0.492405564
ACP1	Acid Phosphatase 1	0.452082694
CTNNA1	Catenin Alpha 1	0.452082694
EHMT2	Euchromatic Histone Lysine Methyltransferase 2	0.452082694
FOXA1	Forkhead Box A1	0.452082694
GKN1	Gastrokine 1	0.452082694
GSK3B	Glycogen Synthase Kinase 3 Beta	0.452082694
HSPB1	Heat Shock Protein Family B (Small) Member 1	0.452082694
KRT14	Keratin 14	0.452082694
NDRG1	N-Myc Downstream Regulated 1	0.452082694
NGF	Nerve Growth Factor	0.452082694
OGT	O-Linked N-Acetylglucosamine (GlcNAc) Transferase	0.452082694
ONECUT1	One Cut Homeobox 1	0.452082694
PCNA	Proliferating Cell Nuclear Antigen	0.452082694
RAC3	Rac Family Small GTPase 3	0.452082694
RHOQ	Ras Homolog Family Member Q	0.452082694
S100A7	S100 Calcium Binding Protein A7	0.452082694
SIRT6	Sirtuin 6	0.452082694
SPHK1	Sphingosine Kinase 1	0.452082694
SRSF3	Serine and Arginine Rich Splicing Factor 3	0.452082694
STK38	Serine/Threonine Kinase 38	0.452082694
TP63	Tumor Protein P63	0.452082694

**Supplementary Table 2:** The expression of all anoikis-related genes

Gene	ConMean	TreatMean	logFC	*p* value	fdr
BRMS1	18.31334	40.09548	1.130545	8.39E-18	8.98E-17
PTK2	4.637303	9.191043	0.986943	7.18E-17	6.70E-16
BCL2L11	3.281152	5.427075	0.725972	9.47E-09	2.35E-08
SRC	9.476183	15.98159	0.754033	2.41E-11	7.98E-11
CEACAM6	120.5597	33.36922	−1.85316	4.15E-13	1.84E-12
CAV1	24.22758	98.38569	2.021798	1.02E-12	4.44E-12
AKT1	8.906661	13.19592	0.567135	5.39E-12	2.04E-11
ITGB1	29.83564	57.29262	0.941313	1.12E-08	2.74E-08
CEACAM5	145.188	28.17943	−2.36521	1.58E-08	3.77E-08
EGFR	15.54844	39.43583	1.342737	5.40E-07	1.06E-06
CASP8	2.141759	3.781403	0.820125	2.05E-11	6.97E-11
PTRH2	2.828445	4.589057	0.698189	4.66E-10	1.35E-09
STAT3	35.27708	36.36855	0.043961	0.654895	0.673394
SIK1	1.175423	1.026727	−0.19513	0.843183	0.85019
TLE1	5.616875	7.83431	0.480038	2.46E-07	5.08E-07
DAPK2	1.002264	0.694241	−0.52975	0.006703	0.009071
CTNNB1	50.68536	51.67402	0.02787	0.913102	0.915617
ZNF304	2.083512	2.433126	0.223793	0.029328	0.037326
BMF	1.414171	2.970118	1.070564	1.09E-09	2.98E-09
MAPK1	11.04435	13.72781	0.313794	9.04E-05	0.000147
ITGA5	4.903082	28.72014	2.550302	9.95E-21	2.01E-19
MCL1	108.2345	114.52	0.081438	0.291929	0.32397
BCL2L1	27.23383	30.72361	0.173948	0.15571	0.181081
CASP3	11.35766	14.67126	0.369327	0.000314	0.000479
BAD	16.34459	24.39265	0.577633	7.51E-07	1.43E-06
PIK3CA	2.380573	4.340237	0.866465	1.41E-11	4.90E-11
PAK1	8.587547	15.70234	0.870662	1.94E-10	5.75E-10
ITGAV	11.75486	28.89517	1.297571	5.61E-14	3.10E-13
FN1	14.90543	134.6144	3.174923	4.79E-16	3.87E-15
MAPK3	26.35448	17.3991	−0.59904	1.37E-10	4.08E-10
PTGS2	2.972568	10.72968	1.851825	0.000572	0.000853
BAX	12.44263	23.10898	0.893162	1.36E-16	1.21E-15
BCAR1	7.399878	11.62259	0.651358	9.21E-10	2.54E-09
PTEN	6.633318	7.598861	0.196052	0.010535	0.013995
ERBB2	25.54295	20.47268	−0.31922	3.47E-10	1.01E-09
PDK4	39.65115	2.901449	−3.77252	1.41E-15	1.01E-14
ANGPTL4	15.65958	19.98927	0.35218	0.220457	0.249212
CYCS	31.68959	34.92588	0.140287	0.277131	0.308488
ANKRD13C	4.193882	4.643341	0.146877	0.031307	0.039707
BRAF	1.952846	2.164484	0.148445	0.329477	0.362325
ANXA5	51.35229	94.19229	0.87518	2.42E-13	1.14E-12
MTOR	5.518004	6.730803	0.286632	0.003123	0.004355
BIRC5	5.051176	20.54713	2.024246	2.54E-24	2.32E-22
TIMP1	95.74281	128.9646	0.429739	5.44E-05	9.09E-05
ITGA2	12.58255	18.35073	0.544413	0.000313	0.000479
CSPG4	4.163772	18.8308	2.177131	2.09E-10	6.13E-10
BSG	101.312	166.4619	0.716388	6.24E-14	3.39E-13
AKT2	7.676326	7.884142	0.038538	0.986085	0.986085
STK11	5.982203	7.778649	0.378843	1.40E-06	2.61E-06
IGF1	0.25138	0.123164	−1.02929	3.06E-06	5.60E-06
IGF1R	4.979949	9.995816	1.005193	7.48E-12	2.70E-11
ITGA6	32.65177	143.8335	2.139167	3.79E-19	5.52E-18
ILK	2.289875	3.004701	0.391952	3.62E-05	6.10E-05
CFLAR	4.929788	4.616782	−0.09464	0.057301	0.070465
RHOA	170.6279	159.1221	−0.10072	0.005931	0.008085
HIF1A	32.81322	67.25712	1.03541	2.96E-12	1.17E-11
DAP3	14.03513	19.78896	0.495653	5.35E-12	2.04E-11
MYBBP1A	10.28701	14.68264	0.513288	1.32E-07	2.80E-07
ITGA3	15.59001	57.98995	1.895181	7.21E-16	5.35E-15
PTK2B	5.230626	8.19616	0.647964	4.48E-07	9.06E-07
CCND1	40.29128	64.57769	0.680568	0.570333	0.598275
CTTN	34.58309	81.80493	1.242121	4.29E-05	7.19E-05
CALR	234.1102	421.6166	0.848744	8.40E-21	1.80E-19
CHEK2	1.75091	4.046747	1.208658	3.21E-15	2.12E-14
SKP2	4.439742	10.44456	1.234203	1.69E-15	1.14E-14
E2F1	2.056468	10.93776	2.411077	1.12E-24	1.36E-22
EGF	1.406144	0.353105	−1.99357	1.03E-05	1.82E-05
PIK3CG	0.423677	0.703268	0.731109	0.118015	0.139472
ITGB4	48.31807	140.246	1.537325	3.69E-18	4.20E-17
PIK3R1	8.780768	4.247492	−1.04774	6.58E-07	1.27E-06
MAP2K1	13.94483	19.01097	0.447102	6.72E-09	1.72E-08
CXCL12	15.01197	5.444742	−1.46318	4.72E-07	9.50E-07
LGALS3	125.5295	88.99508	−0.49623	3.50E-05	5.95E-05
BAK1	14.99677	30.67254	1.032296	1.71E-13	8.55E-13
ABHD4	9.74255	12.269	0.332646	0.233094	0.261871
CD44	58.7771	105.6647	0.846168	4.83E-11	1.50E-10
ITGA4	0.533643	1.252057	1.230354	6.23E-05	0.000103
FADD	3.871827	16.37527	2.080432	3.41E-21	8.88E-20
TGFB1	12.69138	45.45074	1.840455	3.46E-25	1.26E-22
HMCN1	0.867611	1.299774	0.58314	0.668624	0.685575
MMP2	38.69276	78.27592	1.016505	7.98E-09	2.00E-08
CEBPB	29.05665	46.08272	0.665357	4.36E-08	9.85E-08
CDKN3	2.725998	10.06447	1.884414	9.26E-24	5.62E-22
CBL	2.274933	3.687269	0.696728	3.01E-08	6.90E-08
CASP9	2.789836	2.474241	−0.17319	0.025822	0.03333
SFN	2034.71	2527.442	0.312855	0.108271	0.12964
MTDH	16.87518	27.05149	0.680805	1.33E-13	6.91E-13
TNFRSF10B	7.036808	15.80843	1.167701	4.24E-15	2.66E-14
CXCL8	13.85515	50.9419	1.878431	1.02E-07	2.19E-07
AR	0.841429	0.279	−1.59258	2.16E-13	1.04E-12
CDKN2A	1.010931	12.66919	3.647567	8.32E-05	0.000136
PIK3CB	4.650559	5.635174	0.277056	0.102067	0.122615
CPT1A	9.363545	13.53872	0.531965	0.126443	0.148469
MAPK8	2.260326	2.877347	0.348209	0.000116	0.000185
CLDN1	53.72277	116.1745	1.112688	0.000155	0.000242
CDKN1A	91.1972	114.5772	0.329258	0.006928	0.00934
ATF4	103.0126	113.7047	0.142471	0.173561	0.199294
KLF12	0.641811	1.081368	0.752639	0.00024	0.00037
NTRK1	0.058011	0.241851	2.059711	4.82E-14	2.70E-13
MYC	73.5427	72.22842	−0.02602	0.473958	0.505926
PLAU	17.17849	110.7916	2.689173	9.82E-22	2.75E-20
SMAD4	3.646231	3.539153	−0.043	0.160407	0.185949
MUC1	31.19732	15.43909	−1.01483	0.001558	0.002224
PLK1	3.524893	13.33401	1.919459	8.79E-23	2.86E-21
PLAUR	6.597745	16.0868	1.285833	2.62E-13	1.22E-12
LGALS1	109.0298	368.78	1.758038	1.07E-14	6.52E-14
PYCARD	17.38101	31.98672	0.879961	5.27E-08	1.16E-07
KRAS	6.60387	7.920921	0.262357	0.170122	0.196585
BID	3.94089	9.340369	1.244958	6.64E-16	5.04E-15
HRAS	31.41219	45.08741	0.5214	3.57E-05	6.05E-05
CDK11B	6.215619	7.878761	0.342071	2.98E-07	6.08E-07
CDK11A	0.595995	1.011396	0.762976	5.78E-10	1.63E-09
XIAP	6.030716	7.499299	0.314426	0.000936	0.001373
PPARG	2.888424	0.75499	−1.93575	2.08E-17	2.10E-16
IL6	14.19213	5.87052	−1.27353	0.059691	0.072912
CCR7	1.526339	2.867334	0.909634	0.006197	0.008417
RAC1	105.512	131.6608	0.319419	0.000512	0.000769
NOTCH1	10.07847	12.16974	0.272022	0.109591	0.130791
RHOG	32.81732	42.27302	0.36528	6.36E-06	1.14E-05
CCAR2	9.937999	12.43619	0.323517	0.007075	0.009468
NQO1	32.52281	46.23674	0.507588	0.400556	0.436534
BIRC3	4.087472	8.081941	0.983493	0.114541	0.135807
MMP13	0.847039	70.49821	6.379014	1.19E-20	2.28E-19
MTA1	4.318061	7.704283	0.835277	1.05E-13	5.55E-13
MYO5A	2.868487	6.507435	1.181799	6.24E-12	2.33E-11
EDA2R	0.651582	0.319245	−1.02928	2.71E-06	4.99E-06
MMP9	4.682507	79.76467	4.090397	3.49E-23	1.59E-21
ABL1	11.62789	14.91791	0.359456	6.23E-05	0.000103
MAPK11	3.662574	6.672163	0.865297	2.38E-11	7.94E-11
PTHLH	5.480407	63.67381	3.538345	1.19E-18	1.61E-17
PDGFB	3.104907	6.560776	1.079317	1.30E-09	3.52E-09
GLI2	0.643801	1.899178	1.560689	0.021299	0.027689
EZH2	3.171713	6.638469	1.065588	1.20E-08	2.92E-08
CXCR4	9.222526	19.14025	1.053376	0.000385	0.000581
RIPK1	9.502309	10.97815	0.208285	0.017865	0.023476
HMGA1	104.1887	181.4746	0.800569	2.40E-08	5.56E-08
TNFSF10	37.08434	85.98443	1.213266	3.80E-08	8.65E-08
SIK2	5.116516	3.808725	−0.42585	0.000353	0.000535
ANGPTL2	13.82291	17.5634	0.345511	0.18255	0.208956
S100A4	73.64563	71.20226	−0.04868	0.611618	0.632469
ETV4	3.926077	8.900659	1.180824	5.10E-07	1.02E-06
NTF3	0.90704	0.389738	−1.21866	3.23E-13	1.45E-12
HTRA1	30.30233	81.21947	1.422397	2.78E-11	8.96E-11
LATS1	3.250592	4.076164	0.32651	0.001668	0.002372
CEACAM3	0.165774	0.151992	−0.12522	0.110256	0.131154
EIF2AK3	4.39196	4.115332	−0.09386	0.525864	0.558059
LAMC2	16.7289	230.3645	3.783504	3.42E-23	1.59E-21
LAMB3	53.5958	223.739	2.061625	1.29E-18	1.65E-17
LAMA3	5.18223	40.17894	2.954794	1.50E-19	2.38E-18
CDH2	0.27806	1.153357	2.052374	0.007012	0.009418
CSNK2A1	13.76656	18.14122	0.398103	7.01E-09	1.78E-08
EDIL3	1.82772	5.408707	1.565239	2.91E-05	4.98E-05
ZEB2	0.875709	1.047074	0.25784	0.035613	0.044503
TLN1	24.25519	36.83547	0.602802	0.000152	0.000239
EPHA2	73.39707	43.16752	−0.76578	0.000195	0.000304
SIRT3	2.986587	3.072916	0.04111	0.408986	0.441754
CEMIP	5.045043	5.995218	0.248946	0.053249	0.065704
CLU	111.1711	25.97697	−2.09748	5.88E-16	4.55E-15
SPINK1	0.044008	0.413958	3.233664	8.26E-10	2.30E-09
CPEB2	4.878915	4.142881	−0.23593	0.032019	0.040468
NAT1	1.548264	1.23559	−0.32545	0.441969	0.473166
TSG101	17.30285	17.32565	0.001899	0.792857	0.803899
SERPINA1	3.733868	9.802049	1.392412	3.01E-07	6.12E-07
AKT3	1.090395	2.369531	1.11975	5.96E-07	1.16E-06
RELA	17.2217	21.74284	0.336313	7.19E-07	1.38E-06
PRKCA	1.0391	1.818962	0.80778	1.04E-05	1.83E-05
TNFRSF1A	36.22132	48.37269	0.417354	4.64E-09	1.22E-08
FASLG	0.323249	0.723503	1.162353	0.024714	0.032015
AFP	0.007351	0.039632	2.430618	6.42E-05	0.000106
SATB1	2.311661	1.122729	−1.04192	8.91E-11	2.70E-10
EEF1A1	770.3657	582.4162	−0.40349	2.42E-07	5.03E-07
LTB4R2	4.494323	4.119455	−0.12565	0.556873	0.587541
PBK	3.231172	8.524078	1.399486	8.66E-12	3.08E-11
NOX4	0.213872	0.914809	2.096724	7.43E-17	6.76E-16
CD63	132.3195	143.4366	0.116388	0.0357	0.044503
MAVS	9.091915	8.557386	−0.08741	0.058886	0.07217
HRC	15.832	2.455079	−2.689	0.000135	0.000214
PPP1R13B	4.357496	4.232004	−0.04216	0.406728	0.440622
MET	6.42722	16.07803	1.322824	1.98E-13	9.72E-13
RAF1	12.84578	10.65078	−0.27033	2.34E-08	5.47E-08
PARP1	14.6988	26.57501	0.854372	1.10E-14	6.57E-14
BRCA2	0.368605	1.089562	1.5636	1.69E-15	1.14E-14
RB1	10.35047	13.09382	0.339189	0.001372	0.001997
DOCK1	6.467952	7.398461	0.193916	0.044576	0.055189
HAVCR2	1.505471	3.462193	1.201471	8.52E-09	2.12E-08
INHBB	3.192493	3.983541	0.319368	0.187171	0.212907
RANBP9	25.76165	16.33201	−0.65752	8.83E-08	1.91E-07
PDCD4	46.33207	21.70575	−1.09393	4.24E-15	2.66E-14
PRPF4B	7.065991	8.054699	0.188939	0.076818	0.093206
SESN1	5.243547	2.994066	−0.80844	4.58E-08	1.02E-07
SESN3	6.339101	12.12525	0.935664	0.000134	0.000213
PHLDA2	27.32433	32.44653	0.247878	0.013174	0.017438
ZBTB7A	15.02226	13.14049	−0.19308	0.034407	0.043186
CD24	322.2363	139.3393	−1.20952	3.59E-16	3.11E-15
ELANE	0.362007	0.053137	−2.76823	2.24E-11	7.55E-11
MDM2	4.869839	5.64495	0.213087	0.583262	0.608331
KDR	2.639441	2.709828	0.037969	0.171366	0.197397
NFE2L2	49.6146	42.63778	−0.21863	0.029254	0.037326
PRKCI	8.358897	12.9678	0.633549	8.98E-07	1.69E-06
HK2	21.87831	32.42448	0.567582	0.003053	0.004274
KL	0.250263	0.213245	−0.23093	0.003298	0.004583
FGF2	0.889105	0.818794	−0.11885	0.017626	0.023245
EPHB6	7.107745	4.375857	−0.69983	7.00E-08	1.54E-07
CRYAB	167.0406	34.64364	−2.26954	2.03E-09	5.45E-09
LTF	917.6744	35.94535	−4.67411	1.04E-12	4.45E-12
SDCBP	23.51988	28.08224	0.255777	0.000704	0.001046
MGAT5	5.914308	8.103987	0.454422	0.000709	0.00105
SPIB	0.349002	2.855308	3.032338	0.674438	0.689594
PDGFRB	6.27611	17.82387	1.505868	6.29E-12	2.33E-11
TLR3	2.377006	1.523663	−0.6416	2.45E-06	4.52E-06
ROCK1	3.439256	4.891049	0.508047	1.27E-05	2.22E-05
NRAS	12.33457	20.26933	0.716591	4.09E-11	1.29E-10
CASP10	2.160351	2.674425	0.307963	0.026357	0.033901
PLAT	14.00419	16.83782	0.265846	0.084598	0.102305
PAK4	8.152675	10.32428	0.340695	0.001435	0.002064
VEGFA	4.16607	10.37621	1.316521	2.46E-12	9.84E-12
PIN1	6.35804	8.942395	0.492079	2.32E-07	4.85E-07
TWIST1	2.615244	5.401027	1.046288	2.01E-08	4.75E-08
YWHAZ	137.0671	192.0728	0.486771	2.00E-05	3.45E-05
UBE2C	10.39057	45.27313	2.12338	3.49E-24	2.54E-22
BMP6	0.982856	1.148606	0.224831	0.7967	0.805553
ELK1	8.318122	11.98825	0.527292	7.48E-08	1.63E-07
PRDX4	26.52284	40.26961	0.602456	1.47E-08	3.54E-08
BNIP3	7.90732	12.83115	0.69839	0.000114	0.000183
BNIP3L	18.42754	22.56428	0.292177	0.009333	0.012444
KDM3A	3.536094	5.264402	0.574113	9.99E-06	1.78E-05
LMO3	0.198883	0.112174	−0.82618	6.80E-07	1.31E-06
TUBB3	0.359493	1.486287	2.047676	4.63E-16	3.83E-15
SLC2A1	60.52895	234.2819	1.952548	3.51E-18	4.12E-17
PTPN11	14.32772	17.24152	0.267078	0.00128	0.001871
PAK3	0.212204	0.127958	−0.72979	0.004956	0.006781
LRP1	13.33006	19.46389	0.546117	0.00011	0.000177
PIK3R2	0.203212	0.311395	0.615763	3.94E-06	7.13E-06
PTK6	36.58638	16.68792	−1.1325	1.26E-07	2.68E-07
CDH3	32.46422	94.52303	1.541815	1.28E-14	7.49E-14
CASP6	4.109995	6.510983	0.663739	6.48E-10	1.81E-09
CD36	4.521261	2.358752	−0.9387	0.0185	0.024223
EEF2K	5.583433	6.327433	0.180468	0.254866	0.28545
GLO1	40.64781	65.91452	0.697419	2.89E-13	1.31E-12
LPAR1	4.986229	4.342499	−0.19942	0.122052	0.143777
PAK2	13.43084	26.75126	0.994057	7.62E-18	8.41E-17
TRAF2	5.112718	11.24992	1.137752	1.42E-12	5.86E-12
ADCY10	0.012564	0.098291	2.967769	5.21E-08	1.16E-07
CEACAM1	23.81091	4.681894	−2.34646	4.30E-16	3.64E-15
RBL2	7.472103	7.919013	0.083806	0.560895	0.590074
SIRPA	10.44129	20.40543	0.966653	2.01E-09	5.42E-09
TNFRSF12A	17.77236	56.28971	1.663236	3.91E-15	2.54E-14
PIK3R3	2.852351	3.178832	0.156345	0.320172	0.35316
MNX1	0.018929	0.104106	2.45941	5.30E-10	1.51E-09
APOBEC3G	1.428213	3.617965	1.340968	2.52E-07	5.19E-07
BAG1	15.71501	11.91024	−0.39994	4.45E-08	1.00E-07
IL17A	0.166524	0.067734	−1.29778	0.379319	0.41588
COL13A1	0.216707	1.080756	2.318225	9.42E-23	2.86E-21
RAD9A	2.494955	4.797646	0.943313	1.30E-12	5.50E-12
IFI27	39.01783	189.8601	2.282731	2.85E-17	2.80E-16
BCL2L15	0.762143	0.136795	−2.47804	1.60E-15	1.12E-14
SNAI2	9.426433	35.7837	1.924519	5.63E-23	2.28E-21
NOTCH3	26.5488	40.66437	0.615119	0.001444	0.00207
PTPN1	16.33052	26.22633	0.683445	2.80E-13	1.29E-12
MYH9	127.9836	230.4802	0.848684	4.49E-12	1.74E-11
RPS6KB1	4.28172	4.837179	0.175976	0.032259	0.040631
SIRT1	3.624596	3.501049	−0.05003	0.231147	0.260488
PPP2R1A	41.01804	42.89547	0.064567	0.582579	0.608331
MMP11	0.670774	26.53	5.305655	7.32E-25	1.33E-22
COL4A2	12.47388	62.06733	2.314924	7.08E-21	1.61E-19
CD151	38.90278	67.51187	0.795268	1.22E-10	3.68E-10
ARHGEF7	3.293966	4.062065	0.302388	0.000241	0.00037
PPP2R5A	19.17772	16.5087	−0.21621	0.000196	0.000304
BST2	37.59415	254.8219	2.760909	1.32E-18	1.65E-17
PPP2R2D	3.171573	4.479758	0.498222	6.71E-15	4.14E-14
CCDC178	0.07727	0.017247	−2.16357	1.08E-11	3.78E-11
SHC1	21.34367	43.47939	1.026524	5.25E-17	5.03E-16
CDC25C	0.516771	2.219412	2.102581	7.96E-23	2.86E-21
BUB1	1.710408	5.666885	1.728215	6.89E-21	1.61E-19
FER	1.387222	1.790483	0.36815	0.004716	0.006502
ITGB5	16.85811	30.79321	0.86917	7.13E-12	2.59E-11
SETD2	6.83275	6.163023	−0.14883	0.018901	0.024659
TP73	2.293267	4.534157	0.98343	6.07E-06	1.09E-05
BUB3	6.926674	12.65465	0.869433	1.71E-20	3.12E-19
SLCO1B3	0.068606	1.145383	4.061355	6.40E-11	1.96E-10
CDK1	4.731817	14.58463	1.623983	1.83E-20	3.17E-19
MAD2L1	1.966104	5.783662	1.556644	2.50E-19	3.79E-18
BCL2L2	13.45062	14.47302	0.105693	0.380755	0.416201
TDGF1	0.049071	0.038191	−0.36164	0.002382	0.00336
PDCD6IP	20.40737	15.41152	−0.40508	5.27E-07	1.05E-06
SH3GLB1	20.13678	16.61214	−0.27759	5.38E-07	1.06E-06
EDAR	1.496716	0.717442	−1.06087	0.0001	0.000162
DYNLL2	15.39156	15.21008	−0.01711	0.417521	0.449638
TSC2	6.322719	7.11398	0.170112	0.642709	0.662736
MAP3K7	4.586373	6.371236	0.47422	6.69E-12	2.46E-11
BAG4	3.665205	6.3014	0.781778	1.34E-06	2.51E-06
F10	1.573231	0.37288	−2.07695	8.72E-12	3.08E-11
F3	66.70473	69.75341	0.064475	0.876845	0.881689
HSP90B1	94.82423	192.3032	1.020055	5.37E-20	8.89E-19
ADAMTSL1	0.271072	0.241255	−0.16812	0.406728	0.440622
SERPINB1	244.5921	96.74976	−1.33805	2.71E-08	6.23E-08
MAP3K1	5.753347	4.497959	−0.35513	0.00142	0.002059
CTBP1	10.54307	12.70683	0.269309	0.000138	0.000217
CEACAM4	0.316645	0.506744	0.678393	0.000521	0.000781
PXN	10.019	24.16003	1.269883	8.91E-16	6.49E-15
MALAT1	7.392316	11.12716	0.589986	0.002796	0.00393
IKBKG	1.415399	2.336568	0.723183	2.15E-14	1.24E-13
TFDP1	23.83973	36.49752	0.614431	1.10E-07	2.36E-07
CRYBA1	0.019411	0.0461	1.247931	6.72E-05	0.00011
SERPINE1	15.19701	128.4948	3.07985	8.58E-19	1.20E-17
ACTG1	1413.665	1441.546	0.028177	0.486353	0.517638
ARHGDIA	73.53997	100.7372	0.453996	3.50E-09	9.29E-09
EZR	188.4719	167.367	−0.17133	0.18452	0.210549
SLC39A6	18.26425	34.19764	0.904874	4.64E-11	1.46E-10
TIAM1	8.254501	5.602011	−0.55924	0.001435	0.002064
PDPK1	2.547682	2.518078	−0.01686	0.586687	0.610155
SMAD7	3.674136	6.377043	0.795483	7.13E-09	1.80E-08
NTRK3	0.203377	0.040956	−2.312	2.78E-11	8.96E-11
RHOC	23.32079	46.03098	0.980989	9.99E-14	5.35E-13
CASP2	3.633341	5.997788	0.723134	5.83E-09	1.52E-08
TNC	23.51883	115.6361	2.297704	3.63E-12	1.42E-11
IRF6	48.96966	80.54532	0.717913	3.95E-09	1.04E-08
HOTAIR	0.015786	0.299069	4.243791	1.96E-18	2.38E-17
GNE	9.979346	4.104163	−1.28186	3.75E-06	6.83E-06
XAF1	0.786112	3.071366	1.966074	2.10E-12	8.50E-12
SFRP1	31.93154	9.283172	−1.78229	5.34E-11	1.65E-10
MAP2K2	23.03109	25.34606	0.138179	0.21158	0.239923
CSK	21.29519	20.78194	−0.0352	0.603951	0.626319
PIK3C2B	4.860598	3.072133	−0.66189	2.20E-07	4.62E-07
FOXC2	0.480331	2.196439	2.193067	1.07E-08	2.64E-08
TAGLN	28.95494	31.34963	0.114639	0.028235	0.036189
ENDOG	5.263162	5.11662	−0.04074	0.545555	0.577273
ARHGDIB	39.03633	43.80943	0.166424	0.436075	0.468233
FBLIM1	12.32337	29.4257	1.25568	1.65E-13	8.35E-13
CCDC80	11.16683	6.947347	−0.68469	0.319203	0.35316
RACK1	180.8739	165.0623	−0.13197	0.004925	0.006765
PRKD1	0.489846	0.67399	0.460399	0.27101	0.3026
LDHA	112.4477	197.3562	0.811548	2.67E-11	8.75E-11
ANXA2	162.2405	185.6451	0.194413	0.131976	0.154468
SPP1	15.36975	106.3315	2.790404	2.30E-14	1.31E-13
SMARCE1	4.339046	4.790058	0.142665	0.141973	0.165635
QSOX1	24.84684	24.39677	−0.02637	0.755469	0.768131
RBFOX2	6.980204	9.965431	0.513663	5.26E-10	1.51E-09
CDC42	59.05889	70.64329	0.258398	2.87E-05	4.94E-05
MAOA	16.12035	12.17683	−0.40474	0.000768	0.001132
PIP5K1C	6.782737	8.769851	0.370685	1.04E-05	1.83E-05
JUP	372.3741	443.692	0.252806	0.101442	0.122267
ATF2	4.480363	6.447307	0.525081	7.87E-07	1.49E-06
OCLN	2.359423	0.894399	−1.39944	1.37E-12	5.72E-12
CRABP2	250.8156	185.2496	−0.43716	0.061468	0.074831
ID2	15.48896	12.54592	−0.30402	0.043013	0.053436
PITPNC1	3.027077	3.06002	0.015616	0.70824	0.722127
AFAP1L1	2.98005	4.975255	0.739434	5.90E-07	1.15E-06
NGF	0.891798	2.084751	1.225086	1.08E-05	1.89E-05
PCNA	43.90651	103.8344	1.241778	2.06E-17	2.10E-16
GSK3B	7.186319	11.53488	0.682678	1.91E-11	6.57E-11
TP63	31.46696	73.17857	1.217583	2.05E-13	9.96E-13
KRT14	2927.466	5905.31	1.01236	1.65E-05	2.86E-05
SPHK1	7.077903	17.8197	1.332079	5.07E-16	4.01E-15
CTNNA1	57.16702	44.32257	−0.36714	0.00382	0.005288
NDRG1	91.60225	241.6854	1.399675	3.02E-11	9.63E-11
OGT	5.529156	8.215424	0.571276	1.67E-06	3.10E-06
EHMT2	5.868161	9.019899	0.620203	6.26E-09	1.62E-08
RAC3	3.490052	11.18945	1.680818	1.91E-08	4.54E-08
SIRT6	4.728459	7.677568	0.699279	1.58E-13	8.10E-13
ACP1	11.21575	16.36896	0.545438	1.66E-12	6.79E-12
FOXA1	7.238367	3.935025	−0.87929	2.16E-08	5.08E-08
ONECUT1	0.001954	0.036779	4.234257	4.36E-13	1.91E-12
SRSF3	29.34989	35.06791	0.256796	0.001737	0.002461

**Supplementary Table 3:** The mainly potential drugs identified by L1000FWD database

Rank	Drug	Similarity Score	*p* value	q-value	Z-score	Combined score
1	Radicicol	−0.2553	2.26e-11	1.38e-07	1.87	−19.88
2	Dasatinib	−0.2340	5.47e-10	1.36e-06	1.76	−16.33
3	BRD-K85660637	−0.2340	7.50e-10	1.69e-06	1.66	−15.17
4	Mometasone Furoate	−0.2340	8.19e-10	1.70e-06	1.83	−16.61
5	GP-42	−0.2128	8.75e-09	1.04e-05	1.75	−14.11
6	NVP-AUY922	−0.2128	1.34e-08	1.15e-05	1.61	−12.66
7	Tyrphostin-AG-1478	−0.2128	1.57e-08	1.27e-05	1.79	−14.00
8	BRD-K35004659	−0.2128	9.13e-09	1.05e-05	1.73	−13.91
9	Dipivefrine	−0.2128	1.34e-08	1.15e-05	1.80	-14.20
10	NSC-3852	−0.2128	1.57e-08	1.27e-05	1.60	−12.48

## Data Availability

All data used and analyzed in this study are available from the corresponding author on reasonable request.
